# The influence of habitats on female mobility in Central and Western Africa inferred from human mitochondrial variation

**DOI:** 10.1186/1471-2148-13-24

**Published:** 2013-01-29

**Authors:** Valeria Montano, Veronica Marcari, Mariano Pavanello, Okorie Anyaele, David Comas, Giovanni Destro-Bisol, Chiara Batini

**Affiliations:** 1 Dipartimento di Biologia Ambientale, Sapienza Università di Roma, P.le Aldo Moro 5, 00185, Rome, Italy; 2 Dipartimento di Storia, Culture, Religioni, Sapienza Università di Roma, P.le Aldo Moro 5, 00185, Rome, Italy; 3 Istituto Italiano di Antropologia, P.le Aldo Moro 5, 00185, Rome, Italy; 4 Department of Zoology, University of Ibadan, Ibadan, Oyo State, Nigeria; 5 Institut de Biologia Evolutiva (CSIC-UPF), Departament de Ciències Experimentals i de la Salut, Universitat Pompeu Fabra, Doctor Aiguader 88, 08003, Barcelona, Spain; 6 Department of Genetics, University of Leicester, Leicester, LE1 7RH, UK; 7 Current address: Department for Integrative Biology and Evolution, University of Veterinary Medicine, Savoyenstr. 1a, A-1160, Wien, Austria

**Keywords:** Mitochondrial DNA, Migration, Population genetic structure, Bayesian inference, Western Central Africa

## Abstract

**Background:**

When studying the genetic structure of human populations, the role of cultural factors may be difficult to ascertain due to a lack of formal models. Linguistic diversity is a typical example of such a situation. Patrilocality, on the other hand, can be integrated into a biological framework, allowing the formulation of explicit working hypotheses. The present study is based on the assumption that patrilocal traditions make the hypervariable region I of the mtDNA a valuable tool for the exploration of migratory dynamics, offering the opportunity to explore the relationships between genetic and linguistic diversity. We studied 85 Niger-Congo-speaking patrilocal populations that cover regions from Senegal to Central African Republic. A total of 4175 individuals were included in the study.

**Results:**

By combining a multivariate analysis aimed at investigating the population genetic structure, with a Bayesian approach used to test models and extent of migration, we were able to detect a stepping-stone migration model as the best descriptor of gene flow across the region, with the main discontinuities corresponding to forested areas.

**Conclusions:**

Our analyses highlight an aspect of the influence of habitat variation on human genetic diversity that has yet to be understood. Rather than depending simply on geographic linear distances, patterns of female genetic variation vary substantially between savannah and rainforest environments. Our findings may be explained by the effects of recent gene flow constrained by environmental factors, which superimposes on a background shaped by pre-agricultural peopling.

## Background

Understanding how human populations interact and admix is one of the primary aims of human evolutionary genetics. To date, three main factors have been studied in detail which could be possible determinants of gene flow within and among human groups: geography, language and social structure.

Geographical factors have been shown to play an important role in shaping genetic structure, at both inter and intra-continental levels (e.g. [1-7]). Along with the evidence which indicates a geographical continental structure of human populations that is, systematically revealed by the analysis of nuclear loci [2-5], natural barriers have also been indicated as one of the possible elements driving the distribution of human diversity at a local level [6,7].

The relationship between linguistic and genetic diversity has been investigated in numerous studies aimed at understanding how cultural factors may shape gene pools (e.g. [8-10]). Their results highlight a variable degree of correlation, depending not only on the geographic location and scale adopted, but also on the genetic loci analysed when the same set of populations is considered [7,11-13].

Finally, following the seminal study by Seielstad et al. [14], there has been a surge of interest in the role of sex-biased matrimonial mobility, an important aspect of human social structure. In accordance with the prevalence of patrilocal habits, where women move to their husbands households after the marriage, higher female transgenerational migration rates have been inferred at both local and continental level in most populations studied [14-18].

Even though the vast literature accumulated over twenty years (e.g. [8,9]) has produced important insights into the structure of human genetic variation, there are two critical points in the current approaches which need to be adequately considered when planning new research work. Inferences based on extent and patterns of gene flow are usually indirect, being derived from analyses of genetic distances among populations, and assuming simplified migration schemes. This is, in fact, the case of the island model [14,18]. Additionally, the relation between genetic variation and geography has been generally investigated simply by focusing on physical linear distances among populations [3,13,19-21], an approach which might be misleading if we consider how human mobility can be influenced by geographical and environmental barriers or even facilitated by natural corridors on both local and global scales [7,22,23].

In this context, given their high cultural and linguistic diversity and their complex history, African populations probably represent one of the most interesting case studies. Recent studies on large-scale datasets regarding autosomal markers (both STRs and SNPs) support the role of both geography and language in explaining the distribution of genetic variation in Africa [24,25]. Among the four linguistic groups found in the continent, the Niger-Congo includes populations with the widest geographical distribution, spanning from the west to the east and south, and yet the highest common autosomal genetic ancestry (see [24,25], but also [2,4]). This is particularly surprising when considering the complexity of this phylum and its history, for the most part deduced from linguistic data. Due to the uncertain position of Kordofanian languages in the NC tree, the initial centre of diffusion of the phylum is still matter of debate. Ehret (2000) proposed the Nuba Mountains in Sudan, whereas Blench (2006) suggested the Western regions of Africa. On the other hand, the later history of this phylum is generally agreed upon. In summary, around 10–8 thousand years ago (kya), NC languages moved through the savannah of Western Africa, reaching the rainforest 2 ky later. Subsequently, the Bantu languages expanded (5 kya) from Cameroon into the equatorial forest of the Congo, and southward. Finally, they spread to the east (the region of great lakes) and to the south of the rainforest (Angola) around 3kya and from there to the south [26]. However, genetic data indicate that the expansion of Bantu speaking individuals through the African continent could have been more complex than previously thought [27-29] and as also previously pointed out by language and archaeology [30,31].

In this work, we investigated the genetic structure and the patterns of gene flow in a broad dataset (85 populations, 5 typed *ex novo* and 80 collected from the literature) of individuals settled in an area spanning from Central to Western Africa. The populations under study inhabit both the savannah and the rainforest regions, and all speak languages belonging to the Niger-Congo phylum [32] and share traditional patrilocal behaviour, which is here assumed to have been constant through time [33-36]. Therefore, the migration of male individuals should be culturally more limited than females and the analysis of maternal lineages, rather than male-specific and autosomal loci, should allow for the exploration of patterns related to geographical habitat differences and/or linguistic barriers. It is in fact reasonable to expect that female gene flow is the main contributor to gene exchange between populations. In a patrilocal context, if either linguistics or geography is playing a role in structuring genetic variation among the populations under study, this should have left a signature in the distribution of mtDNA variation. On the other hand, when the distribution of male lineages is found to be correlated with linguistic diversity [12,13,21], it is difficult to determine whether such a correlation is a cause or effect of genetic isolation, due to the lack of formal models relating linguistic to genetic evolution. Last but not least, the hypervariable region I of mitochondrial DNA (mtDNA) is at present the only source of information on human genetic variation which provides an adequate genetic coverage of populations settled in the region under study [1,37]. We first explore the distribution of maternal lineages using a new multivariate statistical method (the discriminant analysis of principal components, DAPC; [38]). Thereafter, we compare the fit of three different migration models as descriptors of the relationships among the clusters previously identified, using a Bayesian approach [39-41]. By combining these two methods, our study suggests that the genetic structure of Central and Western African populations may be explained by the effects of recent gene flow constrained by environmental factors, which superimposes on a background shaped by pre-agricultural peopling.

## Results

### Intra-population variation and genetic distances

Intra-population diversity parameters are shown in Table 1. HD ranges between 0.932 in Eviya and 1.000 in Akampka, and MNPD between 6.029 in Sefwi-Wiawso and 10.895 in Orungu. Fu's Fs neutrality test provided large significant negative values for the great majority of populations analysed. Only 7 out of 85 departed from this pattern, five of which were located between Gabon and Congo, the other two being settled in Western Africa (Table 1 and Additional file 1: Table S1).

**Table 1:** Intra-population summary statistics

**Population**	**Abbreviation**	**N**	**K**	**S**	**HD**	**MNDP**	**Fs**	**Fs(p)**
**CENTRAL**								
Bakaka	Bak	50	36	59	0.983 +/− 0.008	9.821 +/− 4.571	−17.339	0.000
Bamileke	Bam	48	36	55	0.988 +/− 0.007	8.108 +/− 3.821	−22.157	0.000
**BatekeN**	**Ban**	**53**	**43**	**59**	**0.988 +/− 0.008**	**8.782 +/− 4.116**	**−24.77**	**0.000**
Bassa	Bas	47	40	61	0.993 +/− 0.006	9.433 +/− 4.408	−24.685	0.000
BatekeS	Bat	50	23	42	0.944 +/− 0.017	6.621 +/− 3.179	−5.416	*0.062*
Benga	Ben	50	26	55	0.952 +/− 0.015	9.922 +/− 4.616	−4.526	*0.094*
**Beti**	**Bet**	**48**	**29**	**52**	**0.968 +/− 0.012**	**8.758 +/− 4.112**	**−9.449**	**0.006**
Foumban	Caf	107	71	67	0.989 +/− 0.003	7.959 +/− 3.728	−24.73	0.000
Wum	Caw	115	63	57	0.983 +/− 0.004	7.519 +/− 3.537	−24.782	0.000
Bankim	Cbt	34	24	44	0.968 +/− 0.017	7.686 +/− 3.673	−9.603	0.001
Duma	Dum	47	29	55	0.973 +/− 0.010	9.258 +/− 4.332	−9.884	0.008
Eviya	Evi	38	16	45	0.932 +/− 0.018	9.135 +/− 4.297	−0.79	*0.523*
Ewondo	Ewd	25	12	37	0.933 +/− 0.023	9.933 +/− 4.701	0.954	*0.676*
Ewondo	Ewo	53	39	54	0.983 +/− 0.008	10.162 +/− 4.716	−20.307	0.000
Fang	Fac	39	27	45	0.965 +/− 0.015	9.501 +/− 4.454	−9.457	0.006
Fang	Fag	66	36	55	0.971 +/− 0.009	8.878 +/− 4.145	−12.994	0.005
Fali	Fal	42	27	43	0.978 +/− 0.009	8.197 +/− 3.878	−9.731	0.003
FulbeC	Fuc	34	26	36	0.975 +/− 0.016	6.674 +/− 3.228	−14.831	0.001
Galoa	Gal	51	27	56	0.965 +/− 0.011	9.001 +/− 4.214	−6.128	0.045
Eshira	Gis	40	25	53	0.970 +/− 0.012	10.077 +/− 4.703	−5.839	0.041
Akele	Kel	48	35	54	0.985 +/− 0.008	9.811 +/− 4.571	−16.756	0.000
Kota	Kot	56	32	59	0.967 +/− 0.010	10.562 +/− 4.885	−8.279	0.022
Makina	Mak	45	27	51	0.962 +/− 0.015	9.306 +/− 4.356	−7.284	0.020
Ndumu	Ndu	39	26	53	0.973 +/− 0.012	9.417 +/− 4.417	−8.013	0.010
Ngoumba	Ngo	44	36	52	0.990 +/− 0.007	8.973 +/− 4.213	−23.106	0.000
Ngumba	Ngu	88	43	57	0.969 +/− 0.007	10.081 +/− 4.655	−14.1	0.003
Nzebi	Nze	63	42	64	0.976 +/− 0.001	8.955 +/− 4.181	−22.917	0.000
Obamba	Oba	47	35	63	0.988 +/− 0.007	9.741 +/− 4.542	−17.487	0.000
Orungu	Oru	20	16	40	0.973 +/− 0.025	10.895 +/− 5.173	−3.53	*0.086*
Punu	Pun	52	35	64	0.982 +/− 0.007	9.123 +/− 4.265	−15.937	0.000
Sanga	San	30	21	36	0.970 +/− 0.016	8.970 +/− 4.250	−5.877	0.022
Shake	Sha	51	34	57	0.973 +/− 0.011	10.194 +/− 4.733	−13.011	0.000
Tali	Tal	20	15	34	0.974 +/− 0.022	6.695 +/− 3.296	−4.77	0.025
Ateke	Tek	54	39	53	0.985 +/− 0.007	9.088 +/− 4.248	−21.957	0.000
Tsogo	Tso	64	33	56	0.961 +/− 0.010	9.058 +/− 4.224	−9.5	0.010
Tupuri	Tup	26	24	53	0.994 +/− 0.013	7.917 +/− 3.804	−15.876	0.000
**WEST-CENTRAL**								
Afaha Obong	Ana	37	31	45	0.989 +/− 0.009	7.137 +/− 3.424	−22.296	0.000
Ediene Abak	Ane	26	23	33	0.988 +/− 0.016	6.252 +/− 3.067	−16.121	0.000
Ikot Obioma	Ani	44	37	48	0.991 +/− 0.007	7.246 +/− 3.451	−25.019	0.000
Efut 1	Efe	49	44	58	0.996 +/− 0.005	8.550 +/− 4.021	−24.807	0.000
Efut 2	Efi	48	39	52	0.991 +/− 0.006	7.566 +/− 3.593	−24.958	0.000
Uwanse	Efo	48	40	55	0.988 +/− 0.009	7.779 +/− 3.686	−24.925	0.000
Akampka	Eka	17	17	33	1.000 +/− 0.020	7.698 +/− 3.775	−11.201	0.000
Calabar	Ekc	28	24	44	0.989 +/− 0.012	7.259 +/− 3.504	−14.509	0.000
Ikom	Eki	38	33	51	0.991 +/− 0.009	7.368 +/− 3.524	−24.653	0.000
Akampka	Ekn	50	47	53	0.997 +/− 0.005	7.169 +/− 3.418	−25.03	0.000
Enchi1	Ghe	20	19	35	0.995 +/− 0.018	7.400 +/− 3.612	−11.922	0.000
Enchi	Ghf	59	46	53	0.988 +/− 0.006	6.965 +/− 3.321	−25.054	0.000
Ho	Ghh	87	54	48	0.984 +/− 0.005	6.294 +/− 3.015	−25.138	0.000
Kibi	Ghk	51	42	53	0.989 +/− 0.007	6.452 +/− 3.104	−25.17	0.000
Afaha Eket	Iae	50	36	48	0.984 +/− 0.007	7.234 +/− 3.446	−23.108	0.000
Awa	Iba	28	24	38	0.987 +/− 0.014	7.241 +/− 3.496	−14.54	0.000
Itam	Ibi	48	42	51	0.994 +/− 0.006	7.113 +/− 3.396	−25.042	0.000
Oku	Ibo	48	39	50	0.988 +/− 0.008	7.662 +/− 3.635	−24.939	0.000
**Idoma**	**Ido**	**37**	**28**	**49**	**0.979 +/− 0.012**	**7.096 +/− 3.407**	**−15.86**	**0.000**
Edienne Ikono	Iei	49	43	55	0.995 +/− 0.005	7.985 +/− 3.774	−24.89	0.000
**Igala**	**Iga**	**41**	**35**	**45**	**0.990 +/− 0.008**	**6.754 +/− 3.249**	**−24.98**	**0.000**
Calabar	Igc	96	69	56	0.988 +/− 0.005	7.435 +/− 3.506	−24.865	0.000
Enugu	Ige	54	45	58	0.992 +/− 0.006	8.117 +/− 3.826	−24.863	0.000
Nenwe	Ign	50	38	50	0.981 +/− 0.011	7.739 +/− 3.666	−24.652	0.000
Ntan Ibiono	Ini	50	38	47	0.988 +/− 0.007	7.177 +/− 3.421	−24.965	0.000
Nnung Ndem	Inn	50	39	53	0.989 +/− 0.006	7.962 +/− 3.763	−24.832	0.000
Oku-Iboku	Ioi	50	36	41	0.985 +/− 0.007	7.225 +/− 3.442	−23.131	0.000
Obong Itam	Ita	50	44	45	0.994 +/− 0.005	7.329 +/− 3.488	−24.999	0.000
Ukpom Ette	Iue	50	42	52	0.993 +/− 0.005	7.701 +/− 3.650	−24.935	0.000
Western Nsit	Iwn	36	26	44	0.975 +/− 0.014	7.187 +/− 3.449	−12.604	0.000
Afaha Okpo	Oao	28	23	38	0.987 +/− 0.013	6.598 +/− 3.212	−13.445	0.000
Afaha Ukwong	Oau	70	47	48	0.987 +/− 0.005	7.409 +/− 3.505	−24.943	0.000
**Tiv**	**Tiv**	**51**	**43**	**55**	**0.992 +/− 0.006**	**8.042 +/− 3.797**	**−24.88**	**0.000**
Yoruba	Yor	34	31	42	0.995 +/− 0.009	6.371 +/− 3.099	−25.145	0.000
**WEST**								
Gb1*	Gb1	50	37	47	0.989 +/− 0.006	6.693 +/− 3.211	−24.988	0.000
Gb2*	Gb2	22	15	35	0.957 +/− 0.028	8.216 +/− 3.961	−2.846	*0.095*
Gb3*	Gb3	62	50	51	0.992 +/− 0.005	8.703 +/− 4.072	−24.756	0.000
Gb4*	Gb4	77	49	56	0.978 +/− 0.007	7.289 +/− 3.450	−24.946	0.000
Gb5*	Gb5	77	49	57	0.976 +/− 0.008	7.378 +/− 3.488	−24.93	0.000
Gb6*	Gb6	58	47	61	0.987 +/− 0.008	7.685 +/−3.634	−24.924	0.000
Gb7*	Gb7	26	20	42	0.969 +/− 0.022	7.520 +/− 3.628	−7.982	0.000
Limba	Lim	67	48	56	0.984 +/− 0.007	6.728 +/− 3.211	−25.085	0.000
Loko	Lok	29	27	45	0.988 +/− 0.011	8.393 +/− 3.989	−15.409	0.010
Mandenka	Mad	78	25	44	0.935 +/− 0.012	6.226 +/− 2.989	−4.59	*0.070*
Mende	Men	55	49	59	0.996 +/− 0.004	8.475 +/− 3.980	−24.805	0.000
Serer	Ser	23	18	36	0.968 +/− 0.026	7.533 +/− 3.650	−6.678	0.000
Temne	Tem	122	77	71	0.989 +/− 0.003	7.787 +/− 3.651	−24.715	0.000
Woloff	Wol	48	39	44	0.991 +/− 0.006	7.622 +/− 3.618	−24.947	0.000

N, number of individuals for each population; K, number of haplotypes; S, number of segregating sites; HD, haplotype diversity; MNPD, mean number of pairwise differences; Fs, Fu's statistic; p, statistical significance (in italics, non-significant). In bold, populations typed in the present study; for additional information, refer to Additional file 1: Table S1. For populations labelled with * please refer to Additional file 1: Tables S1 and original publication for further details.

**Figure 1:**
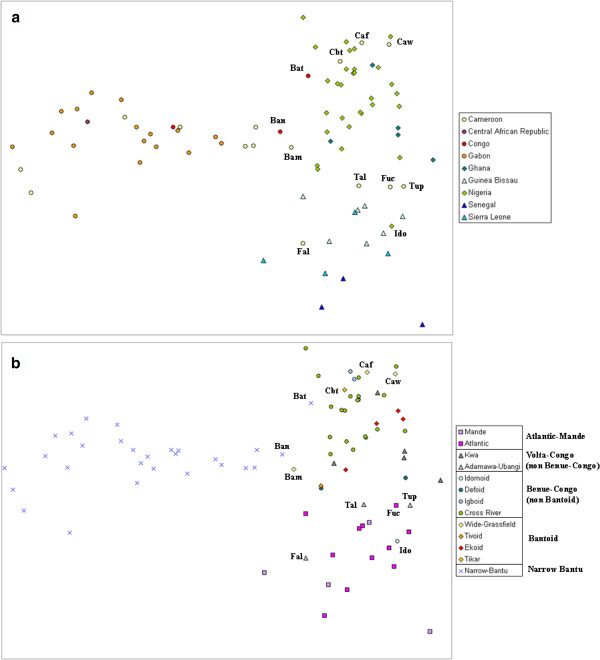
**MDS plot representing a pairwise distance matrix for 85 populations from Central and Western Africa.** Stress value = 0.122. **a**) geographical labels (yellow to orange circles: Central; green diamonds: Central-West; light to dark blue triangles: West) **b**) linguistic labels.

Pairwise genetic distances were calculated among all populations and the matrix represented in a MDS plot, shown in Figure 1. The two-dimensional plot presented a stress value of 0.122, which is lower than the 1% cut-off value of 0.390 ascertained in Sturrock and Rocha (2000) [42]. Populations from Western, Central-Western and Central African regions, are well recognizable in the MDS plot (Additional file 1: Table S1 and Figure 1a), with the latter showing higher average genetic distances. As expected, this geographic trend is no longer observed at single-country level, underlining the non-representativeness of African political boundaries in defining population units. In particular, North Cameroonian populations (Tali, Tupuri and FulbeC) group together with Western populations from Senegal and Sierra-Leone, while Western Cameroonians (Foumban, Wum, Bankim, and, to a lesser extent, Bamileke) are closer to Nigerians and the other Western-Central groups. Both Bateke populations from Congo appeared to be closer to Central Western groups than Central ones. Finally, Idoma from Nigeria present lower average genetic distances from Western African populations than from Western Central, despite their geographical proximity (Additional file 2: Table S2).

From a linguistic point of view (see Figure 1b), the different families of the Niger-Congo phylum already show a geographically structured distribution, but, at a more refined level of classification, linguistic genealogical relationships do not correlate with genetic distances (see Additional file 3: Figure S1b).

### Population genetic structure

The Bayesian Information Criterion (BIC; Additional file 4: Figure S2a) established that 7 was the best number of clusters to describe the genetic structure of the dataset analysed: cluster assignations are presented in Table 2 and Additional file 4: Figure S2b. The *a.score* was 0.752, which means that the probability of re-assignment of populations to true clusters is three times higher than to randomly permuted clusters. Some ambiguity was observed in the population clustering but this mainly concerned pairs of close groups (mostly 3–1; to a much lower extent 2–7 and 5–6, see Additional file 4: Figure S2b).

**Table 2:** **Assignation to DAPC clusters and habitat (s, savannah, and r, rainforest, based on reconstructed map of biomass from Baccini et al., (2008), **[43]**; see** Methods**) for each population with the relative Fu's statistic (Fs) values (in italics, non-significant) and the Fs mean value per cluster**

**Population**	**Country**	**DAPC cluster**	**Habitat**	**Fs**	**Mean Fs**
Ghe	Ghana	1	s	−11.922	
Ghf	Ghana	1	s	−25.054	
Ghh	Ghana	1	s	−25.138	
Ghk	Ghana	1	s	−25.170	
Ghs	Ghana	1	s	−12.390	−21.832
Ibi	Nigeria	1	s	−25.042	
Iga	Nigeria	1	s	−24.979	
Ini	Nigeria	1	s	−24.965	
Ben	Gabon	2	r	*−4.526*	
Evi	Gabon	2	r	*−0.790*	
Ewd	Cameroon	2	s	*0.954*	
Fac	Cameroon	2	r	−9.457	
Gis	Gabon	2	r	−5.839	
Kel	Gabon	2	r	−16.756	−7.388
Kot	Gabon	2	r	−8.279	
Mak	Gabon	2	r	−7.284	
Ngu	Cameroon	2	r	−14.100	
Oru	Gabon	2	r	*−3.530*	
San	Central African Republic	2	s	−5.877	
Sha	Gabon	2	r	−13.011	
Ana	Nigeria	3	s	−22.296	
Ane	Nigeria	3	s	−16.121	
Ani	Nigeria	3	s	−25.019	
Caf	Cameroon	3	s	−24.730	
Caw	Cameroon	3	s	−24.782	
Cbt	Cameroon	3	s	−9.603	
Efe	Nigeria	3	s	−24.807	
Efi	Nigeria	3	s	−24.958	
Efo	Nigeria	3	s	−24.925	
Eka	Nigeria	3	s	−11.201	
Ekc	Nigeria	3	s	−14.509	−21.607
Eki	Nigeria	3	s	−24.653	
Ekn	Nigeria	3	s	−25.030	
Iae	Nigeria	3	s	−23.108	
Iba	Nigeria	3	s	−14.540	
Ibo	Nigeria	3	s	−24.939	
Iei	Nigeria	3	s	−24.890	
Igc	Nigeria	3	s	−24.865	
Ige	Nigeria	3	s	−24.863	
Ign	Nigeria	3	s	−24.652	
Inn	Nigeria	3	s	−24.832	
Ioi	Nigeria	3	s	−23.131	
Ita	Nigeria	3	s	−24.999	
Iue	Nigeria	3	s	−24.935	
Iwn	Nigeria	3	s	−12.604	
Oao	Nigeria	3	s	−13.445	
Oau	Nigeria	3	s	−24.943	
Bak	Cameroon	4	r	−17.339	
Bam	Cameroon	4	s	−22.157	
Ban	Congo	4	r	−24.766	
Bas	Cameroon	4	r	−24.685	−19.009
Bat	Congo	4	r	*−5.416*	
Fal	Cameroon	4	s	−9.731	
Ngo	Cameroon	4	r	−23.106	
Tiv	Nigeria	4	s	−24.877	
Gb5	Guinea Bissau	5	s	−24.930	
Lok	Sierra Leone	5	s	−15.409	
Mad	Senegal	5	s	*−4.590*	−16.893
Men	Sierra Leone	5	s	−24.805	
Ser	Senegal	5	s	−6.678	
Wol	Senegal	5	s	−24.947	
Fuc	Cameroon	6	s	−14.831	
Gb1	Guinea Bissau	6	s	−24.988	
Gb2	Guinea Bissau	6	s	*−2.846*	
Gb3	Guinea Bissau	6	s	−24.756	
Gb4	Guinea Bissau	6	s	−24.946	
Gb6	Guinea Bissau	6	s	−24.924	−18.209
Gb7	Guinea Bissau	6	s	−7.982	
Ido	Nigeria	6	s	−15.857	
Lim	Sierra Leone	6	s	−25.085	
Tal	Cameroon	6	s	−4.770	
Tem	Sierra Leone	6	s	−24.715	
Tup	Cameroon	6	s	−15.876	
Yor	Nigeria	6	s	−25.145	
Bet	Congo	7	r	−9.449	
Dum	Gabon	7	r	−9.884	
Ewo	Cameroon	7	r	−20.307	
Fag	Gabon	7	r	−12.994	
Gal	Gabon	7	r	−6.128	
Ndu	Gabon	7	r	−8.013	−14.052
Nze	Gabon	7	r	−22.917	
Oba	Gabon	7	r	−17.487	
Pun	Gabon	7	r	−15.937	
Tek	Gabon	7	r	−21.957	
Tso	Gabon	7	r	−9.500	

For abbreviations and additional information, refer to Additional file 1: Table S1.

**Figure 2:**
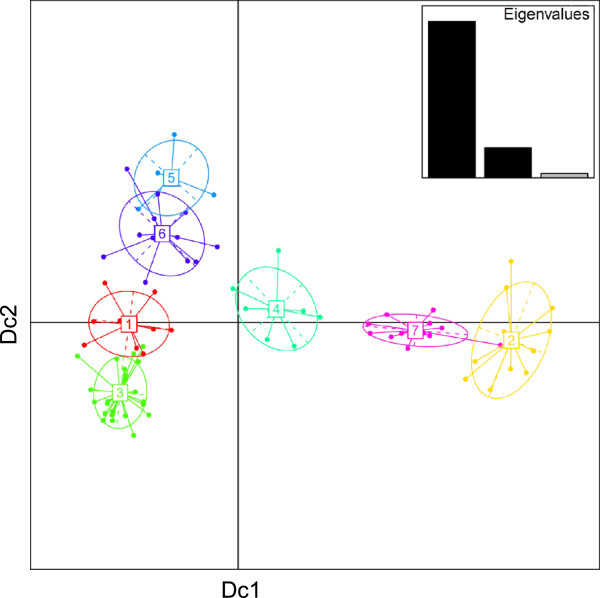
**Scatterplot of the populations' coordinates onto the discriminant functions 1 and 2.** Ellipses of dispersion are proportional to the internal variance of the clusters. In the right upper corner, the eigenvalues for discriminant functions 1 and 2 are reported. See Figure 4 for a map of the populations, labelled according to cluster assignation.

As shown in the bi-dimensional plot, the 7 clusters were distributed according to a geographical pattern (Figure 2). In fact, the first discriminant function separated clusters 4, 7 and 2 (including most of the Central groups) from clusters 5, 6, 1 and 3. The second function separated these last four into two clearly distinguishable groups, a Western (clusters 5 and 6) and a Western-Central one (clusters 1 and 3). The third discriminant function slightly separated cluster 4 and 5 and presented very similar values for the rest (data not shown).

Most clusters were found to group populations that are geographically close together, with few exceptions (see Table 2). Clusters 2, 4 and 7 are composed mainly by populations inhabiting the rainforest areas, starting from Central Cameroon (Table 2, [43]). The less heterogeneous is cluster 4 presenting two populations living in Central-North Cameroon (Bam and Fal) and one population from Nigeria (Tiv). The variance of the geographic distances among clusters was 28 times higher than within cluster (*F* = 28.376, p = 0.000). Cluster 6 was the less geographically homogeneous, including two populations from Nigeria (Yoruba and Idoma) and the three nomadic groups from north Cameroon (Tali, Tupuri and FulbeC) along with Western Africans. On the other hand, the ellipses of dispersion indicated that clusters 3 and 7, even though they account for the highest number of populations, had lower internal variances. This is probably due to the fact that they include the geographical areas with the densest sampling coverage, which results in a higher number of genetically more closely related populations.

Summary statistics calculated for the seven clusters are reported in Additional file 5: Table S3. The MNPD was shown to increase (albeit not significantly) moving from clusters 4, 7 and 2 to the rest. The minimum evolution phylogenetic trees also presented much longer branches and consequently higher divergence for the sequences belonging to cluster 2 and 7 in comparison with the others (Additional file 6: Figure S3). An AMOVA was performed on the rainforest (populations in clusters 4, 7 and 2) vs savannah groups (populations in clusters 5, 6, 1 and 3). The percentage of molecular variance among populations within the two groups was lower than among groups (2.54% vs 5.24%, both p < 0.001).

In addition, the Mantel test showed a low but statistically significant correlation between geographic and genetic distances for the whole sample (r = 0.296; p < 0.001). When dividing the populations according to their habitat, geographic and genetic distances were highly correlated within the savannah region (r = 0.609; p < 0.001), while the rainforest area seemed characterized by a weaker but still significant correlation between the parameters (r = 0.251; p < 0.02). This trend was confirmed when plotting the linear regression for the genetic and geographic distances of the clusters in directions West to East (which implies cluster 5 as the starting point; Additional file 7: Figure S4a) and East to West (with cluster 2 as the point of origin; Additional file 7: Figure S4b). In the former case, the correlation between linear and genetic distances was significant at 0.05 level (*p value* = 0.015) and stronger than in the latter (R^2^ = 0.73 vs R^2^ = 0.53) which was non-significant (*p value* = 0.065).

Interestingly, cluster 2 included four populations with a non-significant value of Fu's statistics. When averaging this parameter among populations within each cluster, cluster 2 presented the least negative value (−7.388), while the others ranged from a mean value of −14.052 to −21.832 (Table 2). The Wilcoxon Mann–Whitney test indicated that the two sets of Fu's values for the savannah and rainforest populations are likely to be drawn from two differing distributions (p-value = 6.817e-06) the median values of the Fu's statistic being −24.794 and −9.499 respectively.

### Migration models and migration rates

**Figure 3:**
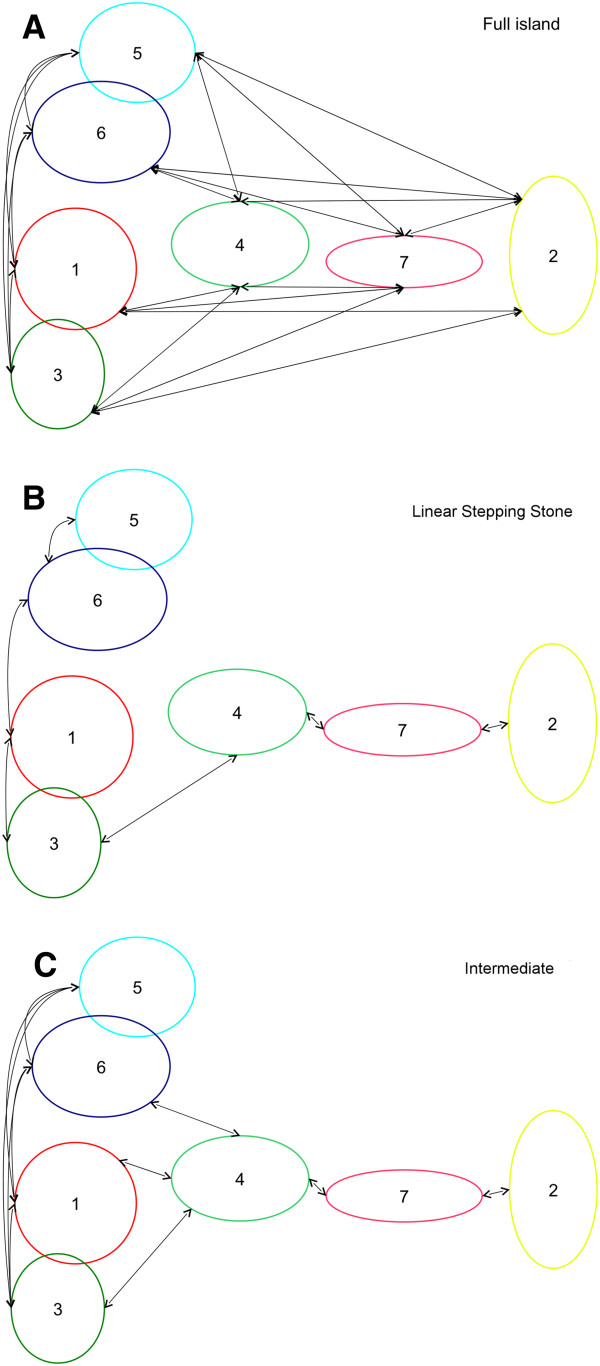
**Schemes of the migration models tested in the present study.A**) Full island. **B**) Linear stepping-stone. **C**) Intermediate (see Materials and Methods for further details).

**Figure 4:**
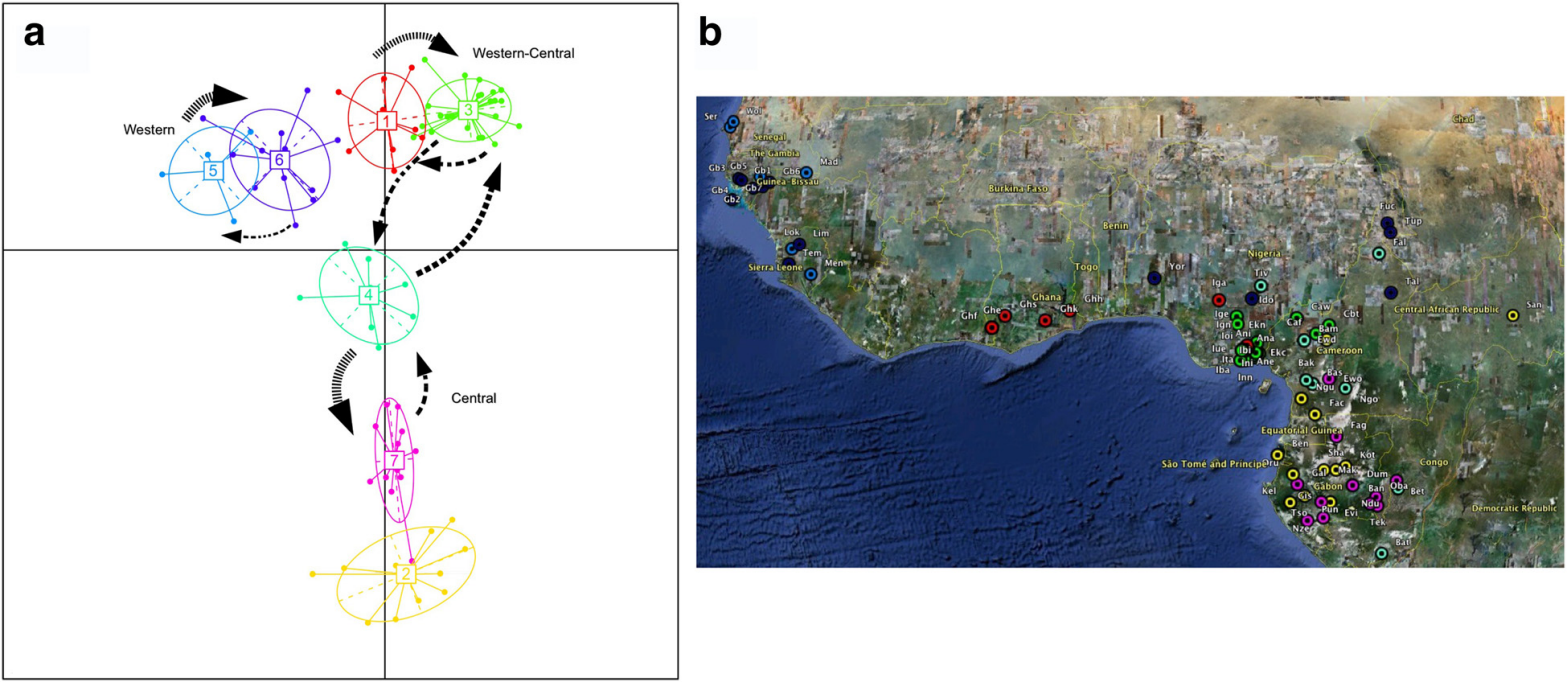
**a) Results of the best migration model among DAPC-clustered populations.** Arrows represent the migration rates > 0.01 and their thickness is proportional to the original value. **b**) Map of the populations labelled according to the cluster analysis with the white lines representing discontinuities in gene flow (see Table 4).

Three different migration patterns were tested through a Bayesian approach, including a full island (A), a linear stepping-stone (B) and an intermediate model (C; see Figure 3 for a schematic representation). The calculation of the LBF indicated model B as the best descriptor for the migration processes occurring in the region under study for all the five independent sub-samples (Table 3; see Material and Methods for details). The values of theta (Θ) and the migration rates (M) obtained with model B were averaged for the fifteen independent runs and are reported in Table 4. Most of the posterior distributions showed normal shapes (Additional file 8: Figure S5) and the runs converged to very close values for all the parameters across the three runs (see standard deviation values in Tables 4 and 5). However, posteriors for the M parameters between clusters 7 and 2 and 1 and 6 were found to have a mode which was close to zero (see Table 4) and a constantly decreasing distribution when moving towards positive values. In these cases, the contribution of migrants exchanged to the observed variation could be considered as null (in italic in Table 4). Therefore, the resulting model is a stepping-stone with two main discontinuities, as described above, across the whole region (Figure 4).

**Table 3:** Log Bayes Factor (LBF) calculated to compare the three migration models

**SUB1**									
LBF (MA | MB)	−714.0927	−868.8034	−959.4728	−821.0730	−761.8231	−826.2525	−873.9830	−847.3129	−954.2933
LBF (MB | MC)	327.0815	360.9551	391.0639	253.9748	434.0619	385.8844	366.1346	439.2414	366.1347
LBF (MA | MC)	−387.0111	−507.8483	−568.4088	−460.1179	−434.7416	−435.1886	−482.9190	−520.2314	−593.3381
SUB2									
LBF (MA | MB)	−1018.3370	−931.4481	−1025.5500	−914.7968	−1034.9890	−967.9367	−984.5880	−1075.9510	−972.4104
LBF (MB | MC)	566.1677	578.1712	629.6136	681.7117	462.6272	680.0142	1388.8972	576.4737	631.3111
LBF (MA | MC)	−452.1696	−353.2769	−395.9367	−336.6256	−468.8209	−338.3231	−354.9744	−509.7832	−394.2400
SUB3									
LBF (MA | MB)	−820.9318	−952.3143	−871.5584	−983.2491	−789.9969	−907.2188	−785.2714	−947.5887	−876.2840
LBF (MB | MC)	285.2062	567.2732	513.2826	404.9559	447.5236	426.9956	371.4934	589.3129	491.2430
LBF (MA | MC)	−535.7255	−385.0410	−358.2758	−415.9758	−504.7906	−393.9361	−500.0651	−380.3154	−363.0013
SUB4									
LBF (MA | MB)	−729.1903	−921.9774	−873.2387	−843.5139	−807.6538	−870.2610	−948.7245	−732.1681	−846.4916
LBF (MB | MC)	130.8856	274.7434	287.9298	160.4199	245.2092	146.8591	261.1827	271.9562	301.4905
LBF (MA | MC)	−598.3048	−647.2339	−585.3090	−568.7705	−676.7682	−582.3313	−660.7947	−601.2825	−571.7482
SUB5									
LBF (MA | MB)	−806.5704	−826.3470	−819.5543	−815.0049	−817.9125	−812.2241	−823.5663	−813.9005	−822.3351
LBF (MB | MC)	521.3028	616.3976	414.1053	607.9631	529.7374	416.8861	408.4515	526.9566	613.6168
LBF (MA | MC)	−285.2675	−209.9494	−405.4490	−198.6073	−296.6097	−398.1189	−409.4610	−292.5977	−205.9374

Each sub-sampling was run three times for each model allowing 27 pairs of model comparisons based on the thermodynamic integration value. MA is the full island model, MB the stepping-stone model and MC is the intermediate model. An LBF > 2 indicates a higher probability for the numerator model; values < 2 indicate the contrary.

**Table 4:** Theta and M values estimated for MB (stepping-stone)

	**Averaged values of theta and M**							
		**M incoming**						
	θ	Clu 2	Clu 7	Clu 4	Clu 3	Clu 1	Clu 6	Clu 5
M outgoing	Clu 2	0.0198	*0.2500*	-	-	-	-	-
	Clu 7	*0.2500*	0.0269	5.2500	-	-	-	-
	Clu 4	-	10.6500	0.0129	7.3500	-	-	-
	Clu 3	-	-	4.8500	0.0198	5.1500	-	-
	Clu 1	-	-	-	8.0500	0.0127	*0.2500*	-
	Clu 6	-	-	-	-	*0.2500*	0.0254	2.7500
	Clu 5	-	-	-	-	-	11.050	0.0067
	s. d. of theta and M values							
	θ	Clu 2	Clu 7	Clu 4	Clu 3	Clu 1	Clu 6	Clu 5
	Clu 2	0.0003	0.0000	-	-	-	-	-
	Clu 7	0.0000	0.0005	0.3535	-	-	-	-
	Clu 4	-	0.2236	0.0003	0.2236	-	-	-
	Clu 3	-	-	0.2236	0.0000	0.4183	-	-
	Clu 1	-	-	-	0.2738	0.0004	0.0000	-
	Clu 6	-	-	-	-	0.0000	0.0005	0.4183
	Clu 5	-	-	-	-	-	0.2738	0.0002

Values of thetas are reported on the diagonal. Direction of migration is represented as outgoing from the clusters in row and incoming into the clusters in column (e.g. M is 5.2500 in the direction 7 - > 4, and 10.6500 in the direction 4 - > 7); "--" states for migration flows not allowed.

**Table 5:** Averaged values of first and last percentile of the distributions of Theta and M with standard deviations calculated combining all the runs for the stepping-stone model

	**2.5%**	**s.d.**	**mode**	**s.d.**	**97.5%**	**s.d.**
θ clust2	0.0055	0.0003	0.0198	0.0003	0.0390	0.0012
θ clust7	0.0103	0.0004	0.0269	0.0005	0.0527	0.0025
θ clust4	0.0000	0.0000	0.0129	0.0003	0.0301	0.0011
θ clust3	0.0058	0.0002	0.0198	0.0000	0.0386	0.0019
θ clust1	0.0000	0.0000	0.0127	0.0004	0.0308	0.0013
θ clust6	0.0095	0.0005	0.0253	0.0005	0.0512	0.0023
θ clust5	0.0000	0.0000	0.0067	0.0002	0.0194	0.0017
M7- > 2	0.0000	0.0000	0.2500	0.0000	10.0000	0.0000
M2- > 7	0.0000	0.0000	0.2500	0.0000	10.5000	0.3535
M4- > 7	0.0000	0.0000	10.6500	0.2236	22.2000	0.5700
M7- > 4	0.0000	0.0000	5.2500	0.3535	15.1000	0.2236
M3- > 4	0.0000	0.0000	4.8500	0.2236	14.5000	0.0000
M4- > 3	0.0000	0.0000	7.3500	0.2236	17.2000	0.2738
M1- > 3	0.0000	0.0000	8.0500	0.2739	17.8000	0.4472
M3- > 1	0.0000	0.0000	5.1500	0.4183	15.2000	0.2738
M6- > 1	0.0000	0.0000	0.2500	0.0000	10.4000	0.4183
M1- > 6	0.0000	0.0000	0.2500	0.0000	9.7000	0.2738
M5- > 6	0.0000	0.0000	11.0500	0.2739	22.4000	0.8944
M6- > 5	0.0000	0.0000	2.7500	0.4183	11.8000	0.2738

Cluster 5 shows the lowest value of effective population size, having Θ = 0.007, while, for the remaining clusters, Θ values range between 0.013 and 0.027 (Table 4). Clusters 7, 3 and 6, which have the highest Θ values, presented the highest rates of immigrants ranging from 8 to 11%. Cluster 4 is characterized by high flows both incoming and outgoing, while cluster 1 exchanges high rates of migrants with cluster 3 but no flow is retrieved with cluster 6. Finally, cluster 5 is connected to cluster 6 through a high outgoing but low incoming migrant rate. This is to be expected considering the lower Θ value compared to the other clusters (Table 4).

## Discussion

Populations speaking languages belonging to the Niger-Congo phylum have been the object of several studies, some of which aimed to assess the patterns associated with the diffusion of Bantu languages [13,21,28,29,44,45]. This is the phylum containing the highest number of languages worldwide and genealogical classification of its families is still under debate [46]. However, there is a consensus on the fact that western Atlantic and Mande are more ancient than central Benue-Congo and Bantu branches, while the emergence of Kordofanian remains unclear [26,46,47]. When autosomal variation is analysed, only a slight substructure among the populations belonging to the entire phylum is observed [25]. By increasing both the number of populations and the geographical coverage, we were able to obtain new insights into the relations among Niger-Congo speakers.

The populations included in our dataset speak languages belonging to several sub-branches of the NC family (see Additional file 3: Figure S1b) and are scattered through a vast area of sub-Saharan Africa, which mainly includes two habitats: the savannah and the rainforest. Roughly speaking, the first prevails in the region from Senegal to Northern Cameroon while the second characterizes most of the areas corresponding to Southern Cameroon, Gabon and Congo. Climatic studies have shown that after the phenomenon known as the Younger Dryas (11.5 ± 0.25 ka B.P; [48,49]), the climatic conditions in the sub-Saharan region became less arid and the distribution and density of the rainforest have remained stable for the last 9.5 ky [50]. The peopling of the sub-Saharan region is likely to have increased since then and the populations here considered have probably been in contact within the same time frame.

Given the shared traditional patrilocal habit of the populations under study, we were able to focus on mtDNA variation as the source of genetic information for microevolutionary inference. By combining a multivariate approach with the test of specific migration patterns, we were able to detect a complex structure among the populations under study, which seems to be better explained by the effect of local environmental factors rather than the internal linguistic complexity of the NC phylum.

After testing three migratory models (Figure 3), we observed that the stepping-stone model better describes the distribution of mtDNA variation throughout the whole region. This may indicate a general tendency of women to spread out from their villages with the intensity of the migration decreasing with distance, so that only neighbouring groups share common genetic variation. The isolation by distance (IBD) pattern observed in our sample is in agreement with previous studies which showed that geographic distances better explain genetic differences among human populations than ethnic affiliations [19,51].

Apart from this general indication, the analysis of mtDNA variation allowed us to identify two main groups quite clearly, with the rainforest populations being more structured and diverse than the savannah groups. In fact, the former populations are characterized by higher values of molecular measures of within-population diversity (see for example the MNPD in Table 1), larger genetic distances and phylogenetic trees with longer branches, and a lower proportion of different haplotypes (corresponding to Central in Table 1, and to clusters 2,7 and 4 in Figure 2). The analysis of genetic structure detected the main signal of differentiation in this group, separating clusters 4, 2 and 7 from the others. The two groups also show a significant difference in the distribution of their Fs values, with rainforest populations showing a less negative average (one tailed *t*-test for mean comparison, p-value = 2.3e-10) as well as including 5 out of the 7 populations with non-significant Fs values (Table 1), suggesting a less important role of demographic expansions in their evolutionary history. The Fu's test, and other statistics relying on haplotype frequencies, were found to be more sensitive for detecting expansions on nonrecombining genomic regions than Tajima's D and other tests [52]. This signature of genetic drift could have been enhanced by the reduced effective population size of the mtDNA compared to autosomal loci, but it is unlikely to have generated the non random genetic structure observed here.

The signature of IBD detected within the savannah region is higher than the one in the rainforest, and indicates, together with the observations of a lower degree of isolation among the former, that the migratory patterns are more straightforward to interpret in the savannah than in the forest. Therefore, we may conclude that although geographic factors have a role in both areas, for the savannah this can be simply described as a linear correlation between physical and genetic distances, while for the rainforest the role played by environmental factors is probably more complex. This conclusion highlights the usefulness of explicit geographic models in trying to understand human genetic diversity, which has been previously suggested by Ray and Excoffier (2009) [53].

As an important evolutionary consideration, we should take into account the possibility that differences in Fu's statistical values between savannah and rainforest could be also explained by the role of selection. However, although the worldwide distribution of mtDNA lineages has been proposed to be driven by selective processes related to temperature changes, the geographic region here analysed appears to be quite homogeneous for this putative temperature effect [54]. In future studies, researchers should consider that other climatic parameters which are different in the savannah and rainforest environments have yet to be explored.

Another caveat of the present study may be the *a priori* definition of population units, based on the sampling location and the languages spoken by the individuals. We are aware that such a definition may lead to an approximation in the estimate of the spatial distribution of allele frequencies, since each population is considered as a sampling point. In the present case, we believe that, despite the vast geographical area covered by our dataset, the homogeneous nature of sampling helps overcoming this limitation and is allowing a reliable representation of the distribution of maternal lineages.

The complexity of the migratory patterns observed here is further emphasized by a discontinuity detected between clusters 7 and 2, which overlaps with a broad area of the rainforest region (encompassing Cameroon, Gabon, Congo and Central African Republic) where the sampling coverage is fairly homogeneous. Cultural factors do not seem to offer an explanation for this separation. In fact, the populations composing the two clusters speak languages that are closely related, within the Narrow Bantu family and show no major differences in their subsistence economy. On the other hand, environmental factors could have played a role if one considers that the rainforest habitat may decrease the intensity of gene flow among populations after their initial settlement in deforested areas, making migration more difficult. Another discontinuity in the pattern (between clusters 1 and 6) overlapped with a gap in the sampling coverage of the dataset under study, corresponding to the area of Guinea, the Ivory Cost and Liberia, where tropical rainforest vegetation generally prevails. In the absence of these samples, any further inference on the validity of the observed discontinuity would be very speculative. However, their analysis could contribute to a more exhaustive testing of the influence of different environments on the intensity of migrations among human populations.

Considering all the previous observations, we suggest that farming rainforest populations have probably undergone a local, more recent, and less intense demographic expansion than other food producer populations of the Niger-Congo phylum, which has been previously observed in Gabon through the analysis of Y chromosome lineages [29]. Evidence of ancient peopling should also be taken into account when interpreting genetic data. In fact, central Africa is characterized by a well-defined succession of Middle Stone Age industries while western Africa seems to have been populated at very low densities until 10–12 kya [47,55]. Rainforest farmers have also been shown to share both recent and ancient genetic backgrounds with hunter-gatherer populations [56-60].

It is interesting to note the unexpected association observed in cluster 6 where populations of nomadic shepherds from Northern Cameroon (Tali, Tupuri and FulbeC; see also MDS plot in Figure 1) were grouped together with Western groups. Complex relationships among Cameroon ethnic groups have already been reported in previous studies [21,24,29,61]. Although the intermediate model we tested was not the best supported by the analysis, it actually detected high migration rates from cluster 6 to clusters 3 and 4 (data not shown). Mixed hierarchical models of migration combined with a better knowledge of the nomadic routes followed by these populations would be worth investigating in order to clarify our findings.

Focusing on the genetic variation of Niger-Congo-speaking populations, our results highlighted a stronger structure among the populations settled in the Central area, which correspond to the Bantu-speaking groups. In fact, populations settled in Nigeria and Ghana (clusters 3 and 1) and Guinea Bissau and Senegal (clusters 6 and 5), which present a high linguistic diversity, seem to be characterized by a rather continuous gene flow and show smaller inter-population differences. This contradicts the expectations described above, based on linguistic data, of a recent demic expansion from the area of Nigeria-Cameroon towards Central Southern and Eastern Africa, and an earlier diffusion from Western to Central Africa [26].

As a general conclusion, language does not seem to be the main predictor for the distribution of genetic variation among Niger-Congo-speaking populations. Despite the general belief that language is transmitted by migrating women, genetic analyses have repeatedly shown its preferential correlation with paternal rather than maternal genetic variation [12,13,21].

Unfortunately, we were unable to find a reliable approach for the definition of linguistic distances. Comprehensive classifications based on a quantitative measure of lexical similarities are only available for the Narrow Bantu languages, and not systematically for other Niger-Congo branches (Koen Bostoen, personal communication). Since in this dataset only 28/85 populations belong to the Narrow Bantu family, we decided to avoid this approach in order not to introduce interpretation biases due to inaccurate or questionable linguistic classification.

Even though the genetic clusters here reported cannot be considered as random mating units, the picture presented in our study suggests that, in particular thanks to female-biased movements, gene flow occurs among human populations speaking very different languages.

The analysis of paternal patterns of migration would be useful to shed light on the substructure and the random mating areas among patrilocal populations, while autosomal and X-chromosomal data could be productively investigated to explore whether sex-biased movements are detectable in the distribution of genome variation.

## Conclusions

In this paper, we present a genetic study on female patterns of migration in populations from Central and Western Africa which share a patrilocal tradition and belong to the same linguistic phylum. Our results show how macro habitats seem to play a major role in determining population genetic structure. Population samples from Guinea, the Ivory Coast and Liberia could allow us to test whether this working hypothesis applies to an even larger area of the continent. However, we highlight here how fundamental the knowledge of cultural factors is when planning a population genetic study. In fact, having reliable information about matrimonial behaviour, even the resolution provided by a relatively small region of mtDNA, proved useful in inferring complex patterns of migration and isolation.

## Methods

### Sampling and database

Our dataset contains 4175 individuals from 85 Niger-Congo speaking populations from Western-Central sub-Saharan Africa (15 Cameroon, 1 Central African Republic, 1 Congo, 17 Gabon, 5 Ghana, 7 Guinea Bissau, 27 Nigeria, 4 Sierra Leone, 3 Senegal; see Additional file 1: Table S1 for further details and Additional file 3: Figure S1a for exact geographical locations). Eighty were obtained from a systematic mining of mtDNA online databases [62] and from current literature, while the remaining 5 were analysed for this study. A total of 230 samples were collected from 3 Nigerian populations (37 Idoma, 41 Igala and 51 Tiv) and 2 Congolese populations (53 North Bateke and 48 Beti). The map of biomass reconstructed by Baccini et al., (2008) [43] was used to assign each population to the savannah or the rainforest group (see Table 2). The threshold for an area to be defined forest is 112 or more of biomass index [43]. Linguistic affiliation, which was defined according to Ethnologue's classification, is reported in Additional file 1: Table S1 ([63] Ethnologue: SIL International. Online version: http://www.ethnologue.com/), while a tree representing structure within Niger-Congo and relations among languages spoken in the populations analysed is presented in Additional file 3: Figure S1b. Sample collection methodology and the aims of the study have been approved by the ethical committees of the University of Ibadan and Sapienza University of Rome. The sampling took place in hospitals under the supervision of the local medical staff in compliance with the Helsinki Declaration. Each participant signed an informed consent which was drafted in English. The forms included the following information: 1) aims, procedure and scientific benefits, absence of economical benefits; 2) the fact that potential injuries related to withdrawal of the check swabs would be treated by the medical staff; 3) personal information about the volunteer is not transferred in digital format and stored as physical brochure; 4) participants can withdraw at any moment; 5) no material is stored in biobanks.

The HVR1 of mtDNA, from position 16024 to 16383, was sequenced in all individuals and used for all further analyses. Sequencing was carried out according to Vigilant et al. (1989) [64], with minor modifications. HVR1 was amplified using primers L15996 and H16401, and then sequenced on both strands using the BigDye Terminator v3.1 Cycle Sequencing Kit (Applied Biosystems). The quality control of the final data was performed through a phylogenetic approach and each missing diagnostic mutation or private change was confirmed through resequencing. Haplogroup assignment was carried out manually and labelling was performed in agreement with PhyloTree [65]. The haplotypes and haplogroups for the newly typed populations are provided in Additional file 9: Table S4. Haplogroup frequencies for the 85 populations included in the study are reported in Additional file 10: Table S5.

### Statistical analyses

Intra-population diversity parameters, Fu's neutrality test, pairwise genetic distances, AMOVA and Mantel test statistics were calculated using Arlequin 3.5 software [66]. The distance matrix was represented in a non-metric multidimensional scaling (MDS) plot using the SPSS 15.0 software (SPSS for Windows, Rel. 11.2006. Chicago: SPSS Inc). A Wilcoxon Mann–Whitney test was used to compare two sets of Fu's statistical values and was performed with an R base package (r-base-core; R Core Development Team 2011; [67]).

Genetic structure was inferred through the Discriminant Analysis of Principal Components (DAPC; [38]). To analyse population structure with mtDNA, we used the matrix of mtDNA mutation frequencies calculated at population level. In this way, all the variation in the individual sequences is included, and the principal components (PC) naturally retrieve the correlation among the variables. Applying the PC analysis directly to individual mtDNA sequences would otherwise have detected the pattern of phylogenetic relationships among the haplogroups [68].

The first step of the structure analysis consisted in assigning populations or individuals to clusters through the *k-means* approach, which relies on classical ANOVA. This method maximizes the variance among groups and minimizes the variance within groups. The Bayesian Information Criterion (BIC) was used to detect the best number of groups comparing the decrease of the residual variance among different numbers of clusters, with the best number corresponding to the minimum BIC value [38].

DAPC was performed on the clusters inferred with the *k-means* in order to investigate their separation which is summarized by the discriminant components [38]. This analysis is composed by a first step, a classical PC analysis, and a second step, which is the actual discriminant analysis applied to the matrix of principal components. The components, or *discriminant functions*, thus maximize the ratio of the variance among groups and the variance within groups. Group positions, defined by the discriminant functions, are presented in a scatterplot. The residual of the probability of population assignment to true clusters versus randomly permuted clusters (*a.score*) was calculated to test the goodness-of-fit of the discriminant analysis [67,69,70].

A simple linear regression analysis was performed to evaluate the correlation between genetic and geographic distances among the clusters using the geographic coordinates of their centroids (calculated as mean(lat) and mean(long) of the populations in the cluster). This was then plotted for both East to West and West to East directions [67].

Mega 5.05 software was used to calculate the alpha value of the gamma distribution for the mutation rate of the whole dataset and to obtain trees of Minimum Evolution for the sequences included in each cluster (see Supplementary Materials for further details; [71]).

Once the unbiased structure of the populations under study was determined, the migration pattern among the clusters identified was tested through a Bayesian approach, which is implemented in migrate-n software version 3.2.9 [39,41]. The software also allows maximum likelihood inference to be drawn, but Bayesian estimation was seen to be more efficient when using data from a single locus [40]. Three migration schemes were modelled and compared, with the aim of explaining the distribution of the clusters in the DAPC plot integrated with their geographical relative locations. The first (model A) is a full island model where all the clusters are allowed to interchange migrants and can be considered as a null model without prior assumptions. The second (model B) is a linear stepping-stone model where cluster 2 and 5 are at the extremes. This is the most parsimonious model allowable, where the connections among the clusters are assigned taking into account both their positions on the discriminant axes and the geographical region most represented in each cluster. The last one (model C) is intermediate between a stepping-stone (Central clusters: 2, 7 and 4) and a full island model (Central-Western and Western clusters: 4, 3, 1, 6 and 5), where cluster 4 represents the link between the two schemes. In model C, we excluded the connection between clusters 4 and 5, since they do not share any population from a common region and they are also separated by the third discriminant component (data not shown). The rationale for the intermediate model is based exclusively on the pattern highlighted in the the DAPC plot. Here, cluster 2 is very well separated from cluster 4, suggesting no close migratory relation and an overall stepping stone model for cluster 4, 7 and 2. The best model was chosen through the Log Bayes Factor (LBF) calculation, which was carried out using the value of thermodynamic integration instead of the harmonic mean, since the latter has been shown to be less reliable [40,72]. The parameters estimated are theta (Θ) and migration rates (M) expressed as the number of migrants. Model details and specific run conditions are provided in a supplementary text.

In order to reduce the prohibitive computational time, migration estimates were carried out on a proportional sub-sampling of each cluster. A random sub-set accounting for 30% of each cluster, for a total of 1024 individuals, was pooled five times. Considering the high amount of samples included in cluster 3 and the fact that they belong to a very small geographical area, which is overrepresented in comparison to the rest of the region, the cluster 3 sampling was reduced to 15% in order to obtain a comparable sample size for all clusters. Each model was then run 3 times for each different sub-dataset for a total of 45 runs. Log Bayes Factors were calculated as follows for a total of 45 crossed comparisons among pairs of models:

Log BayesFactor = 2ln (Prob(D|Model1)-Prob(D|Model2)

Sub-samples were compared with the original sample through basic summary statistics using Arlequin 3.5 software [65]. Comparisons among original clusters and relative sub-samplings for gene diversity and mean number of pairwise differences were found to be non-significant, as well as the F_ST_ values among each cluster and its subsets (Additional file 5: Table S3 and data not shown). The number of polymorphic sites showed a decrease in 10-20% of the original value, which is to be expected given that this statistic is directly dependent on the sample size. Although this does not influence the estimates of theta (Θ) values, the loss of rare haplotypes in the sub-samples may lead to underestimated migration rates. For this reason, instead of calculating the number of immigrants (2 Nm), we discuss the M value which represents the immigration rates scaled for the mutation rate per site per generation (m/μ) and which indicates the relative contribution of migration over mutation processes to the variation observed.

## Competing interests

The authors declare no competing interests.

## Authors' contributions

VaM, CB, GDB, MP designed the research. VeM, CB, DC conceived and designed the experiments. VaM, VeM, OA provided the samples. DC contributed with reagents and materials. VeM performed the experiments and built the database. VaM analysed the data. VaM and CB wrote the paper with the contribution of GDB. All authors read and approved the final manuscript.

## Supplementary Material

Additional file 1: Table S1 Database used in the present study. The populations are listed in a geographical order from East to West with their linguistic affiliation according to ethnologue.com. Click here for file

Additional file 2: Table S2 Pairwise genetic distances matrix among populations. Non-significant distances are reported as null. Click here for file

Additional file 3: Figure S1 a) Map of geographical positions of the 85 populations analysed in the present study: central (green), central-west (red), west (blue). b) Phylogenetic relationships among the languages spoken by the 85 populations analysed in the present study graphically reproduced according to ethnologue.com. Click here for file

Additional file 4: Figure S2 a) Curve of BIC decreasing in relation to number of clusters considered. The minimum BIC value corresponds to number of clusters = 7. b) Assignation of the populations to the clusters. The intensity of the colour is proportional to probability of assignation. Click here for file

Additional file 5: Table S3 Summary statistics for the 45 sub-datasets compared with the original sample belonging to each of the 7 clusters individuated. N is the number of individuals, K the number of haplotypes, k/N is ratio between the two previous values, S is the number of segregating sites and %S the percentual retain of variability in comparison with the original sample. HD is the haplotype diversity, MNPD the mean number of pairwise differences. Click here for file

Additional file 6: Figure S3 Minimum Evolution tree topologies for the 7 DAPC clusters (see Supplementary Material for further details). The tree for cluster 3 was divided across 9 pages for a better graphical visualization. Click here for file

Additional file 7: Figure S4 Plot of the linear regression between genetic vs geographic distances based on the clusters' centroids. a) The linear distances are calculated starting from cluster 5 in direction West to East. b) The linear distances are calculated starting from cluster 2 in direction East to West. Click here for file

Additional file 8: Figure S5 Posterior distributions of the parameters estimated with migrate-n (θ and M) for one of the 15 runs. Click here for file

Additional file 9 Table S4 Haplotypes found in the populations typed in the present study. Click here for file

Additional file 10 Table S5 Frequencies of the main haplogroups in the populations analysed. Click here for file
