# Supplementary figures and images for: The influence of habitats on female mobility in Central and Western Africa inferred from human mitochondrial variation
Source: BMC Evol Biol. 2013 Jan 29;13:24. doi: 10.1186/1471-2148-13-24 (PMC3605107; doi:10.1186/1471-2148-13-24)

A

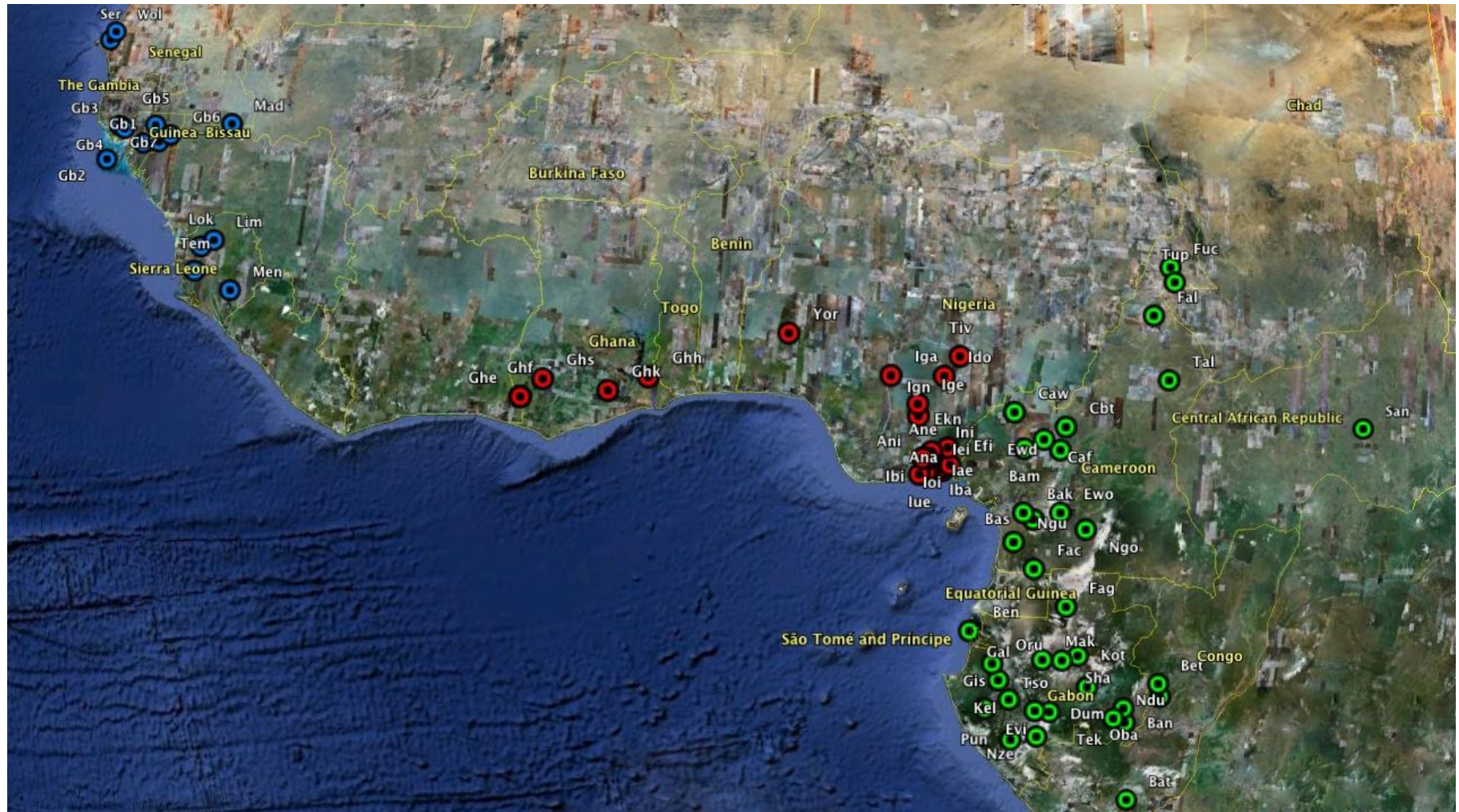

B

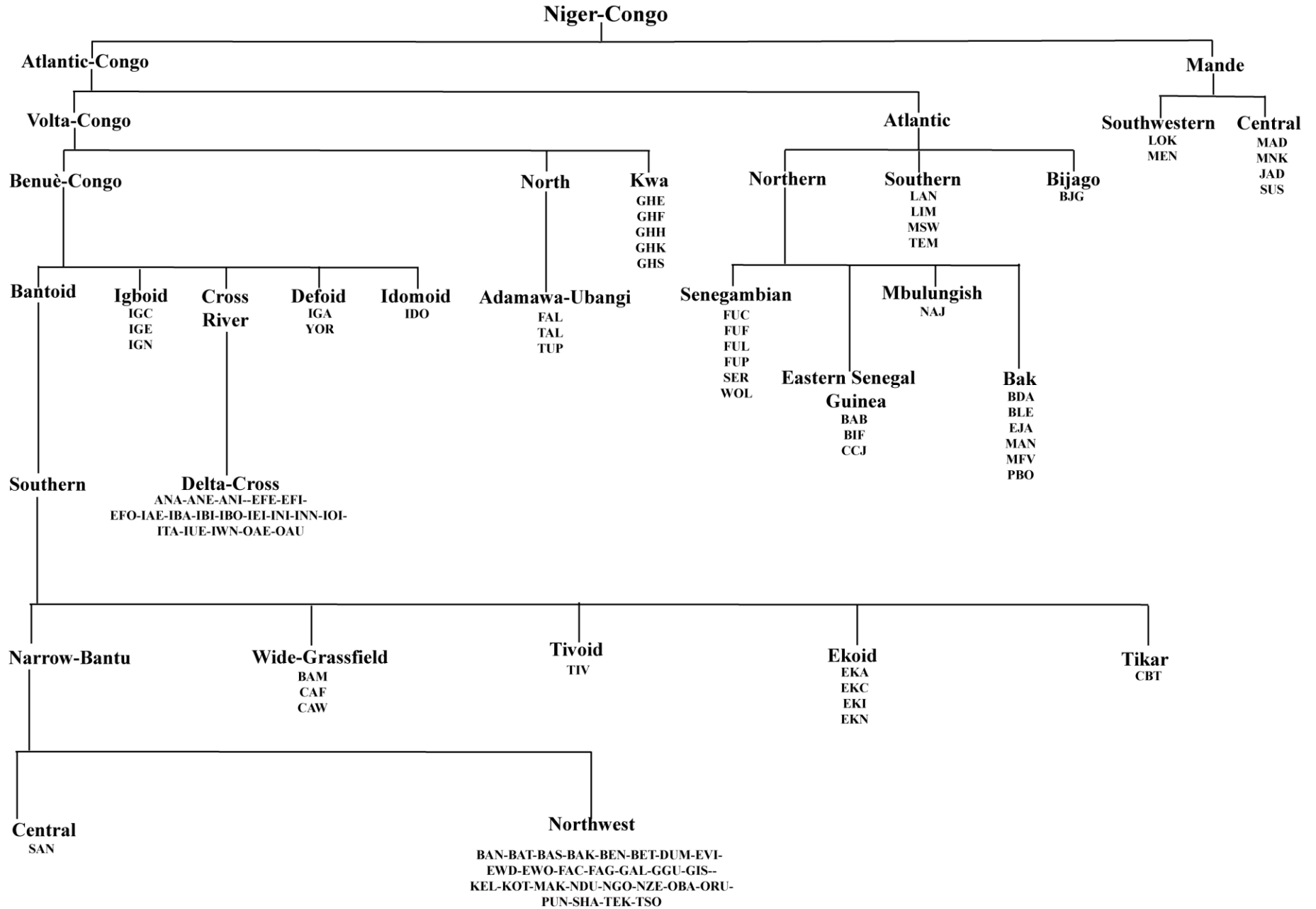

Supplement: Additional file 3: Figure S1 — a) Map of geographical positions of the 85 populations analysed in the present study: central (green), central-west (red), west (blue). b) Phylogenetic relationships among the languages spoken by the 85 populations analysed in the present study graphically reproduced according to ethnologue.com. [file 1471-2148-13-24-S3.pdf]

A

Value of BIC  
versus number of clusters

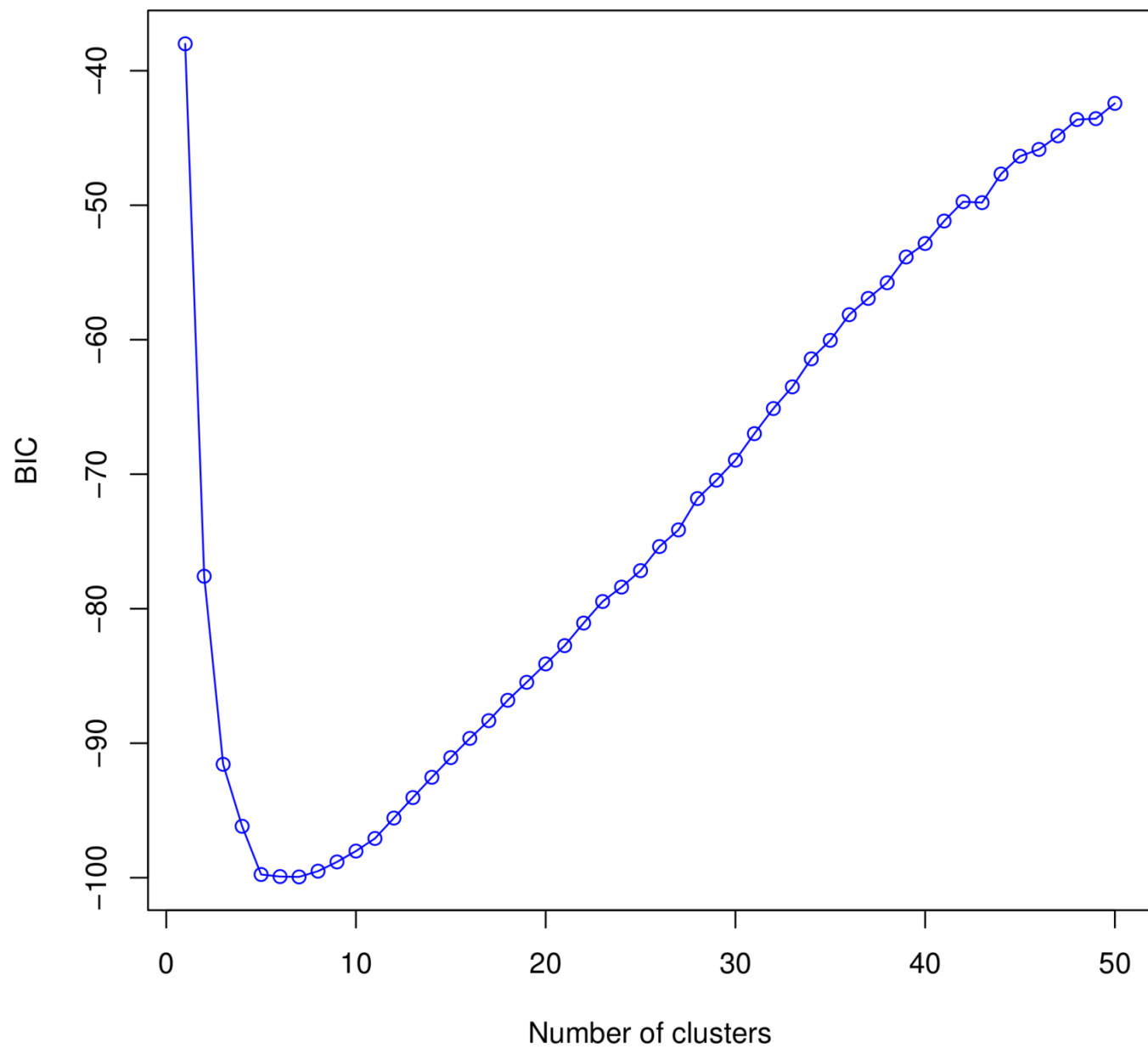

B

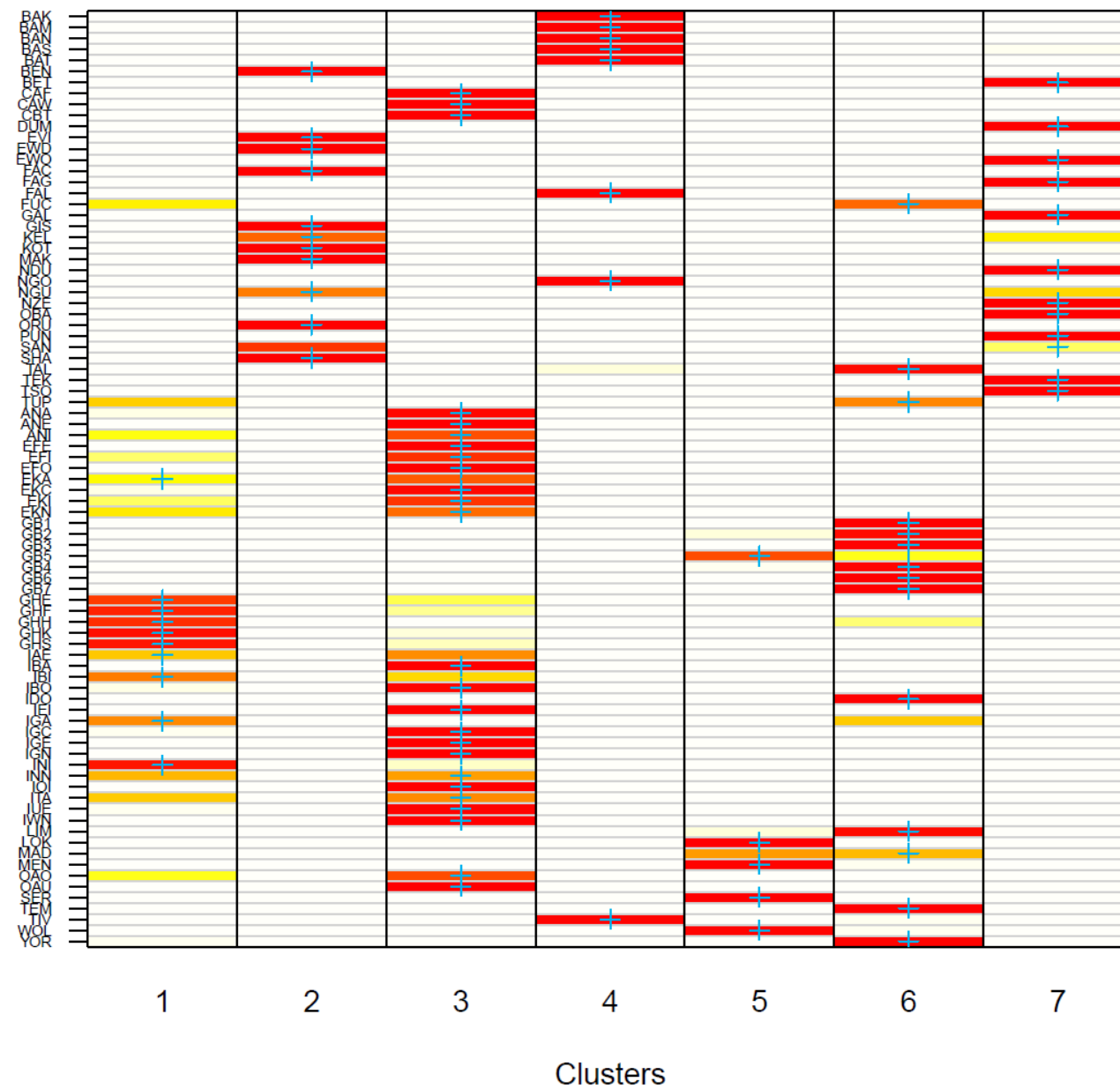

Supplement: Additional file 4: Figure S2 — a) Curve of BIC decreasing in relation to number of clusters considered. The minimum BIC value corresponds to number of clusters = 7. b) Assignation of the populations to the clusters. The intensity of the colour is proportional to probability of assignation. [file 1471-2148-13-24-S4.pdf]

A

WEST to EAST

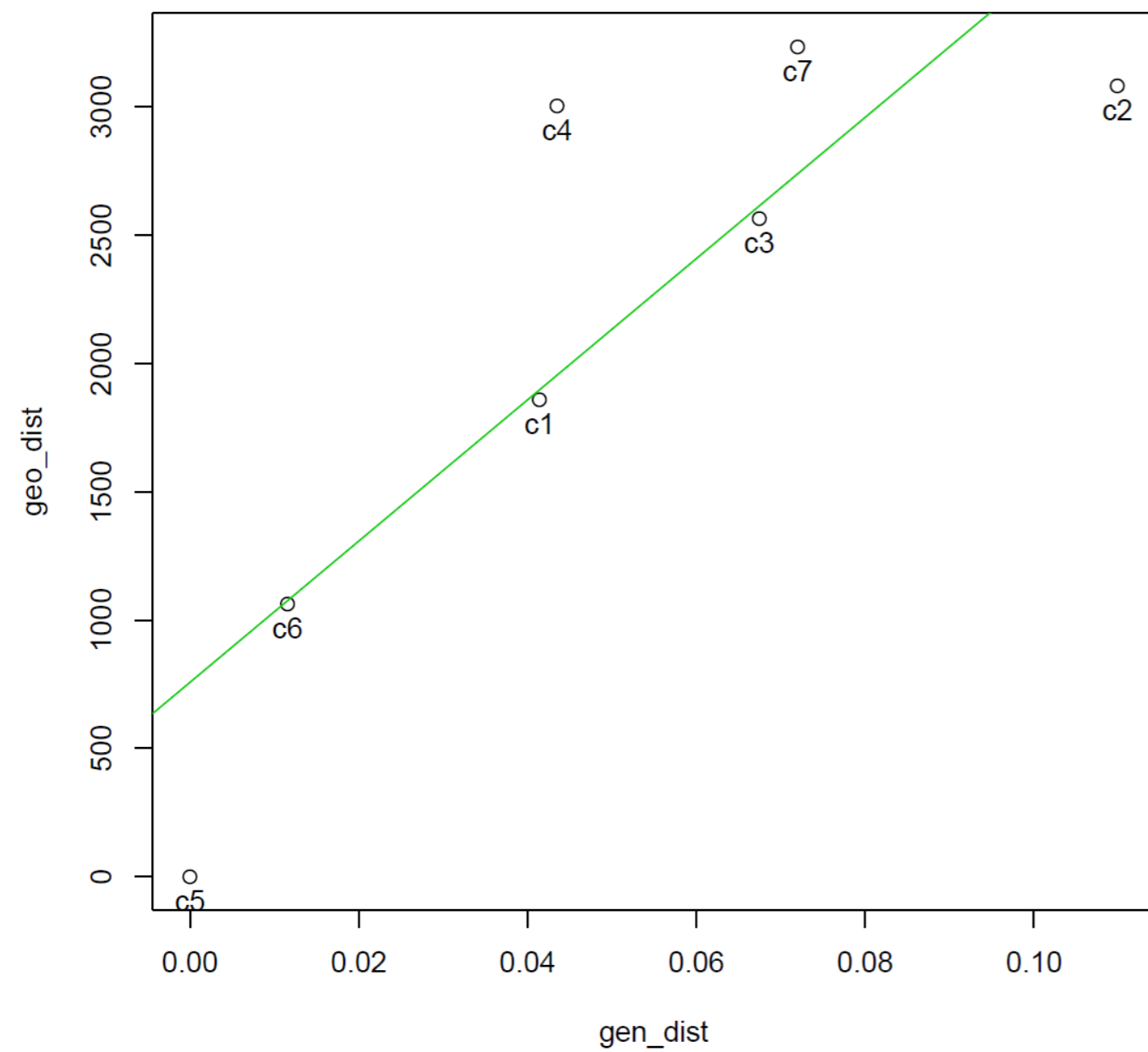

B

EAST to WEST

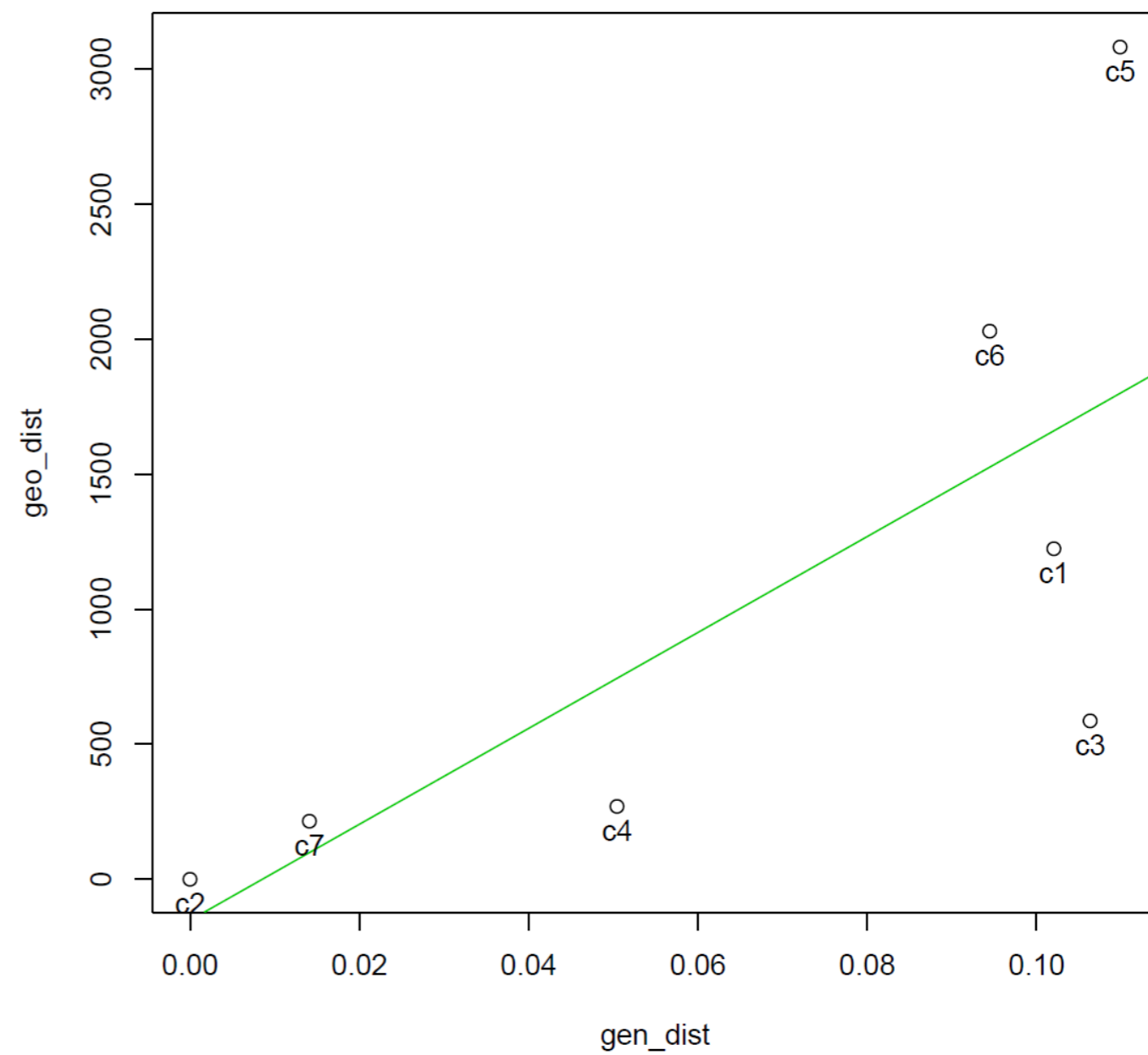

Supplement: Additional file 7: Figure S4 — Plot of the linear regression between genetic vs geographic distances based on the clusters' centroids. a) The linear distances are calculated starting from cluster 5 in direction West to East. b) The linear distances are calculated starting from cluster 2 in direction East to West. [file 1471-2148-13-24-S7.pdf]
