# Supplementary material for: The influence of habitats on female mobility in Central and Western Africa inferred from human mitochondrial variation
Source: BMC Evol Biol. 2013 Jan 29;13:24. doi: 10.1186/1471-2148-13-24 (PMC3605107; doi:10.1186/1471-2148-13-24)
Supplement: Additional file 8: Figure S5 — Posterior distributions of the parameters estimated with migrate-n (θ and M) for one of the 15 runs. [file 1471-2148-13-24-S8.doc]

**1 Run conditions with migrate-n**

| Prior distribution for mutation-scaled population size Uniform 0.000001 1.0 |
| --- |
| Prior distribution for migration rates Uniform 0.000000 500.0 |
| Long-chains 1.0 |
| Long-inc 1,000.0 |
| Long-sample 200.0 |
| Burn-in 200,000.0 |
| Replicates 50.0 |
| Heating (6 chains) 1.00, 1.25, 2.50, 7.50, 15.00, 1000000.00 |

| Mutation model HKY85-model |
| --- |
| Transition/transversion ratio 2.00 |
| Rates 0.34, 1.51, 3.69, 7.41 |
| Rate probabilities 0.54, 0.41, 0.05, 0.00079 |
| Proposal posterior distribution Slice-sampling |

**2. Mutation rate estimate and phylogenetic analysis with Mega 5.05**

The estimated value of the shape parameter for the discrete Gamma Distribution is 0,3807. Substitution pattern and rates were estimated under the Hasegawa-Kishino-Yano (1985) model (+Gamma) [1]. A discrete Gamma distribution was used to model evolutionary rate differences among sites (4 categories, [+G]). Mean evolutionary rates in these categories were 0,01, 0,17, 0,71, 3,11 substitutions per site. The nucleotide frequencies are A = 34,24%, T/U = 21,67%, C = 33,04%, and G = 11,05%. For estimating ML values, a user-specified toplogy was used. The maximum Log likelihood for this computation was -7809,746. The analysis involved 3640 nucleotide sequences. All positions containing gaps and missing data were eliminated. There were a total of 307 positions in the final dataset. Evolutionary analyses were conducted in MEGA5.05 [2]
