# Supplementary material for: The influence of habitats on female mobility in Central and Western Africa inferred from human mitochondrial variation
Source: BMC Evol Biol. 2013 Jan 29;13:24. doi: 10.1186/1471-2148-13-24 (PMC3605107; doi:10.1186/1471-2148-13-24)

Cluster 1

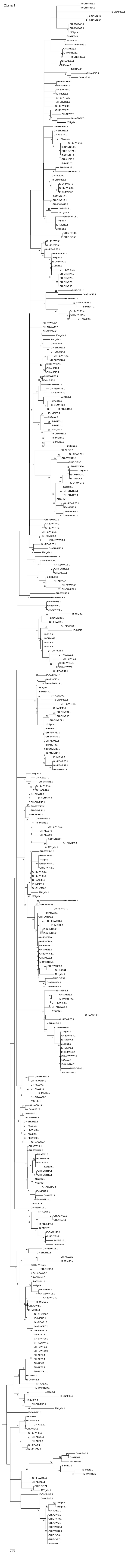

Cluster 2

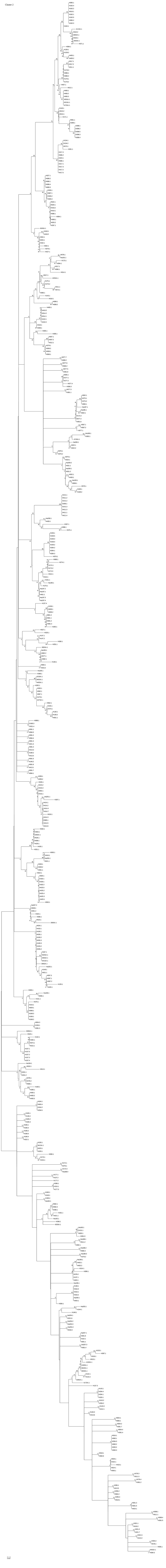

Cluster 3

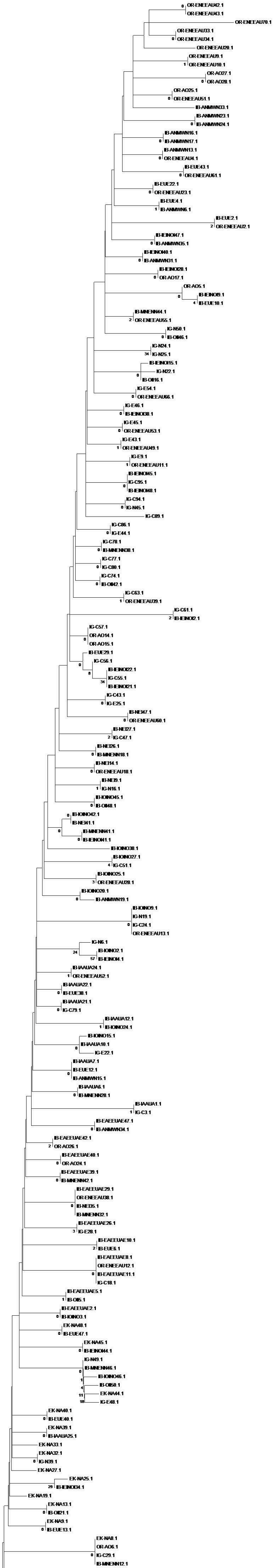

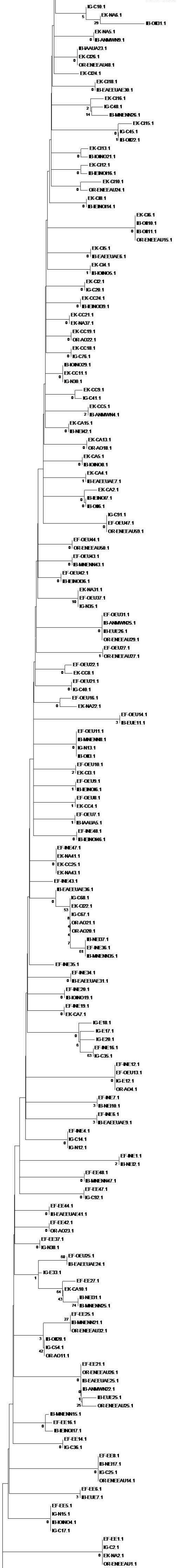
IG-C10.1
EK-NA6.1
IB-OII31.1
EK-NA5.1
IB-ANMWN9.1
IB-IAAUA23.1
EK-CI25.1
OR-ENEEAU48.1
EK-CI24.1
EK-CI18.1
IB-EAEEUAE30.1
EK-CI16.1
IG-C48.1
IB-MNENN26.1
EK-CI15.1
IG-C45.1
IB-OII22.1
EK-CI13.1
IB-IOINO21.1
EK-CI12.1
IB-IEINOI16.1
EK-CI10.1
OR-ENEEAU24.1
EK-CI8.1
IB-IEINOI14.1
EK-CI6.1
IB-OII10.1
IB-OII11.1
OR-ENEEAU15.1
EK-CI5.1
IB-EAEEUAE6.1
EK-CI4.1
IB-IOINO5.1
EK-CI2.1
IG-C20.1
EK-CC24.1
IB-IEINOI39.1
EK-CC21.1
EK-NA37.1
EK-CC19.1
OR-AO22.1
EK-CC18.1
IG-C76.1
IB-IOINO29.1
EK-CC11.1
IG-N30.1
EK-CC9.1
IG-C41.1
EK-CC5.1
IB-ANMWN4.1
EK-CA15.1
IB-NEII42.1
EK-CA13.1
OR-AO18.1
EK-CA5.1
IB-IOINO8.1
EK-CA4.1
IB-EAEEUAE7.1
EK-CA2.1
IB-IEINOI7.1
IB-OII6.1
IG-C91.1
EF-OEU47.1
OR-ENEEAU59.1
EF-OEU44.1
OR-ENEEAU50.1
EF-OEU43.1
IB-MNENN43.1
EF-OEU42.1
IB-IEINOI36.1
EK-NA31.1
EF-OEU37.1
IG-N35.1
EF-OEU31.1
IB-ANMWN25.1
IB-EUE26.1
OR-ENEEAU29.1
EF-OEU27.1
OR-ENEEAU27.1
EF-OEU22.1
EK-CC8.1
EF-OEU21.1
IG-C40.1
EF-OEU16.1
EK-NA22.1
EF-OEU14.1
IB-EUE11.1
EF-OEU11.1
IB-MNENN8.1
IG-N13.1
IB-OII3.1
EF-OEU10.1
EK-CI3.1
EF-OEU9.1
IB-IEINOI6.1
EF-OEU8.1
EK-CC4.1
EF-OEU7.1
IB-IAAUA5.1
EF-INE48.1
IB-IEINOI46.1
EF-INE47.1
EK-NA41.1
EK-CC25.1
EK-NA43.1
EF-INE43.1
IB-EAEEUAE36.1
IG-C68.1
EK-CI22.1
IG-C67.1
OR-AO21.1
OR-AO20.1
IB-NEII37.1
EF-INE36.1
IB-MNENN35.1
EF-INE35.1
EF-INE34.1
IB-EAEEUAE31.1
EF-INE20.1
IB-IOINO19.1
EF-INE19.1
EK-CA7.1
IG-E18.1
IG-E17.1
IG-E20.1
EF-INE16.1
IG-C35.1
EF-INE12.1
EF-OEU13.1
IG-E12.1
OR-AO4.1
EF-INE7.1
IB-NEII10.1
EF-INE6.1
IB-EAEEUAE9.1
EF-INE4.1
IG-C14.1
IG-N12.1
EF-INE1.1
IB-NEII2.1
EF-EE48.1
IB-MNENN47.1
EF-EE47.1
IG-C92.1
EF-EE44.1
IB-EAEEUAE41.1
EF-EE42.1
OR-AO23.1
EF-EE37.1
IG-N38.1
EF-OEU25.1
IB-EAEEUAE24.1
IG-E33.1
EF-EE27.1
EK-CA10.1
IB-NEII31.1
IB-MNENN25.1
EF-EE25.1
IB-MNENN21.1
OR-ENEEAU32.1
IB-OII28.1
IG-C54.1
OR-AO11.1
EF-EE21.1
OR-ENEEAU26.1
IB-EAEEUAE25.1
IB-ANMWN22.1
IB-EUE25.1
OR-ENEEAU25.1
IB-MNENN15.1
EF-EE16.1
IB-IEINOI17.1
EF-EE14.1
IG-C36.1
EF-EE8.1
IB-NEII17.1
IG-C25.1
OR-ENEEAU14.1
EF-EE6.1
IB-EUE7.1
EF-EE5.1
IG-N15.1
IB-IOINO4.1
IG-C17.1
EF-EE1.1
IG-C2.1
EK-NA2.1
OR-ENEEAU1.1

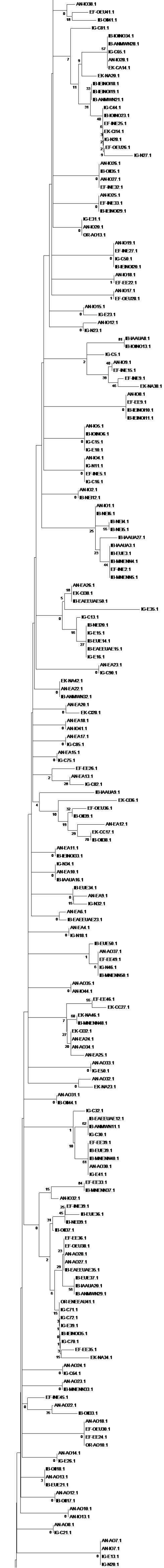

AN-IO38.1
EF-OEU41.1
IB-OII41.1
IG-C81.1
IB-IOINO34.1
IB-ANMWN28.1
IG-C65.1
AN-IO28.1
EK-CA14.1
EK-NA20.1
IB-IEINOI18.1
IB-IEINOI19.1
IB-ANMWN21.1
IG-C44.1
IB-IOINO23.1
EF-INE25.1
EK-CI14.1
IG-N28.1
EF-OEU26.1
IG-N27.1
AN-IO26.1
IB-OII35.1
AN-IO27.1
EF-INE32.1
AN-IO25.1
EF-INE33.1
IB-IEINO29.1
IG-E31.1
AN-IO20.1
OR-AO13.1
AN-IO19.1
EF-INE27.1
IG-C50.1
IB-IEINO20.1
AN-IO18.1
EF-EE22.1
AN-IO17.1
EF-OEU28.1
AN-IO15.1
IG-E23.1
AN-IO12.1
IG-N23.1
IB-IAAUA8.1
IB-IOINO13.1
IG-C5.1
AN-IO9.1
EF-INE15.1
EF-INE9.1
EK-NA38.1
AN-IO8.1
EF-EE9.1
IB-IEINOI10.1
IB-IEINOI11.1
AN-IO5.1
IB-IOINO6.1
IG-C15.1
IG-E10.1
AN-IO4.1
IG-N11.1
EF-INE5.1
IG-C16.1
AN-IO2.1
IB-NE312.1
AN-IO1.1
IB-NE36.1
IB-NE34.1
IB-NE35.1
IB-IAAUA27.1
IB-IAAUA3.1
IB-EUE3.1
IB-MNENN4.1
EF-INE2.1
IB-MNENN5.1
AN-EA26.1
EK-CI38.1
IB-EAEEUAE50.1
IG-E35.1
IG-C13.1
IB-NE120.1
IG-E15.1
IB-EUE14.1
IB-EAEEUAE15.1
IG-E16.1
AN-EA23.1
IG-C90.1
EK-NA42.1
AN-EA22.1
IB-ANMWN32.1
AN-EA20.1
EK-CI28.1
AN-EA18.1
AN-IO41.1
AN-EA17.1
IG-C85.1
AN-EA15.1
IG-C75.1
EF-EE26.1
AN-EA13.1
IG-C82.1
IB-IAAUA9.1
EK-CI36.1
EF-OEU36.1
IB-OII39.1
AN-EA12.1
EK-CC17.1
IB-OII38.1
AN-EA11.1
IB-IEINOI33.1
IG-N34.1
AN-EA10.1
IB-IAAUA16.1
IB-EUE34.1
AN-EA9.1
IG-N32.1
AN-EA6.1
IB-EAEEUAE23.1
AN-EA4.1
IG-N18.1
IB-EUE50.1
AN-AO37.1
EF-EE49.1
IG-N46.1
IB-MNENN50.1
AN-AO35.1
AN-IO44.1
EF-EE46.1
EK-CC27.1
EK-NA46.1
IB-MNENN48.1
EK-CI32.1
AN-EA24.1
AN-AO34.1
AN-EA25.1
AN-AO33.1
IG-E50.1
AN-AO32.1
EK-NA23.1
AN-AO31.1
IB-OII44.1
IG-C32.1
IB-EAEEUAE12.1
IB-ANMWN11.1
IG-C30.1
EF-EE39.1
IB-EUE39.1
IB-MNENN40.1
AN-AO30.1
IG-E41.1
EF-EE33.1
IB-MNENN37.1
AN-IO32.1
EF-INE39.1
IB-EUE36.1
IB-NE39.1
IB-OII37.1
EF-EE36.1
EF-OEU38.1
AN-AO28.1
AN-AO27.1
IB-EAEEUAE35.1
IB-EUE37.1
IB-IAAUA20.1
IB-ANMWN29.1
OR-ENEEAU41.1
IG-C71.1
IG-C72.1
IG-E39.1
IB-IEINOI35.1
IG-C70.1
EF-EE35.1
EK-NA34.1
AN-AO24.1
IG-C64.1
AN-AO23.1
IB-MNENN33.1
EF-INE45.1
AN-AO22.1
IB-OII33.1
AN-AO18.1
EF-OEU30.1
EF-EE24.1
OR-AO18.1
AN-AO14.1
IG-E26.1
IB-OII18.1
AN-AO13.1
IB-EUE21.1
AN-AO12.1
IB-OII17.1
AN-AO10.1
AN-IO13.1
AN-AO8.1
IG-C21.1
AN-AO7.1
AN-IO7.1
IG-E13.1
IG-N20.1

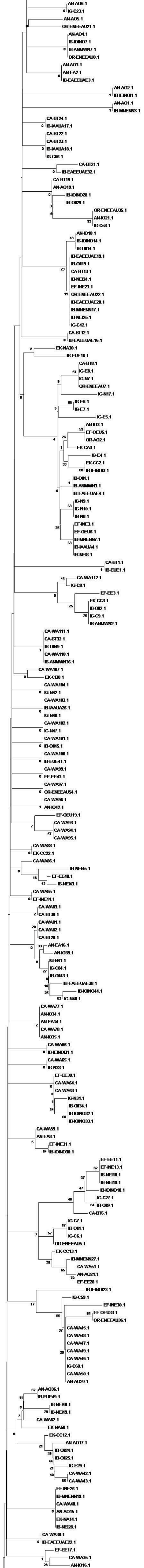

AN-AO6.1
IG-C23.1
AN-AO5.1
OR-ENEEAU21.1
AN-AO4.1
IB-IOINO7.1
IB-ANMWN7.1
OR-ENEEAU8.1
AN-AO3.1
AN-EA2.1
IB-EAEEUAE3.1
AN-AO2.1
IB-IEINOI1.1
AN-AO1.1
IB-MNENN3.1
CA-BT24.1
IB-IAAUA17.1
CA-BT22.1
CA-BT23.1
IB-IAAUA18.1
IG-C66.1
CA-BT21.1
IB-EAEEUAE32.1
CA-BT19.1
AN-AO19.1
IB-IOINO28.1
IB-OII29.1
OR-ENEEAU35.1
AN-IO21.1
IG-C58.1
AN-IO10.1
IB-IOINO14.1
IB-OII14.1
IB-EAEEUAE19.1
IB-OII19.1
CA-BT13.1
IB-NE24.1
EF-INE23.1
OR-ENEEAU22.1
IB-EAEEUAE20.1
IB-MNENN17.1
IB-NE25.1
IG-C42.1
CA-BT12.1
IB-EAEEUAE16.1
EK-NA30.1
IB-EUE16.1
CA-BT8.1
IG-E8.1
IG-N7.1
OR-ENEEAU7.1
IG-N17.1
IG-E6.1
IG-E7.1
IG-E5.1
AN-IO3.1
EF-OEU5.1
OR-AO2.1
EK-CA3.1
IG-E4.1
EK-CC2.1
IB-IEINOI3.1
IB-OII4.1
IB-ANMWN3.1
IB-EAEEUAE4.1
IG-N9.1
IG-N10.1
IG-N8.1
EF-INE3.1
EF-OEU6.1
IB-MNENN7.1
IB-IAAUA4.1
IB-NE8.1
CA-BT1.1
IB-EUE1.1
CA-WA112.1
IG-C8.1
EF-EE3.1
EK-CC3.1
IB-OII2.1
IG-C9.1
IB-ANMWN2.1
CA-WA111.1
CA-BT32.1
IB-OII49.1
CA-WA110.1
IB-ANMWN36.1
CA-WA107.1
EK-CD8.1
CA-WA104.1
IG-N42.1
CA-WA103.1
IB-IAAUA26.1
IG-N48.1
CA-WA102.1
IG-N47.1
CA-WA101.1
IB-OII45.1
CA-WA100.1
IB-EUE41.1
CA-WA99.1
EF-EE43.1
CA-WA97.1
OR-ENEEAU54.1
CA-WA96.1
AN-IO42.1
EF-OEU19.1
CA-WA93.1
CA-WA94.1
CA-WA95.1
CA-WA88.1
EK-CC22.1
CA-WA86.1
IB-NE45.1
EF-EE40.1
IB-NE43.1
CA-WA85.1
EF-INE44.1
CA-WA83.1
CA-BT30.1
CA-WA81.1
CA-WA82.1
CA-BT28.1
AN-EA16.1
AN-IO39.1
IG-N41.1
IG-C84.1
IB-OII43.1
IB-EAEEUAE38.1
IB-IOINO44.1
IG-N40.1
CA-WA77.1
AN-IO34.1
AN-EA14.1
CA-WA78.1
AN-IO35.1
CA-WA66.1
IB-IEINOI31.1
CA-WA65.1
IG-N33.1
EF-EE30.1
CA-WA64.1
CA-WA63.1
IG-N31.1
IB-OII34.1
IB-IOINO32.1
IB-IOINO33.1
CA-WA59.1
AN-EA8.1
EF-INE31.1
IB-IOINO30.1
EF-EE11.1
EF-INE13.1
IB-NE18.1
IB-NE19.1
IB-IOINO10.1
IG-C27.1
IB-OII9.1
CA-BT6.1
IG-C7.1
IB-OII1.1
IG-C6.1
OR-ENEEAU5.1
EK-CC13.1
IB-MNENN27.1
CA-WA51.1
AN-AO21.1
EF-EE28.1
IB-IEINOI23.1
IG-C59.1
EF-INE30.1
EF-OEU33.1
OR-ENEEAU36.1
CA-WA45.1
CA-WA48.1
CA-WA47.1
CA-WA49.1
CA-WA46.1
IG-C60.1
CA-WA50.1
AN-AO20.1
AN-AO36.1
IB-EUE49.1
IB-NE48.1
IB-NE49.1
CA-WA62.1
EK-NA59.1
EK-CC12.1
AN-AO17.1
IB-OII24.1
IB-OII25.1
IG-E29.1
CA-WA42.1
CA-WA43.1
EF-INE26.1
IB-MNENN19.1
CA-WA40.1
AN-AO15.1
EK-NA14.1
IB-NE28.1
CA-WA38.1
IB-EAEEUAE22.1
EF-EE17.1
CA-WA35.1
AN-IO16.1

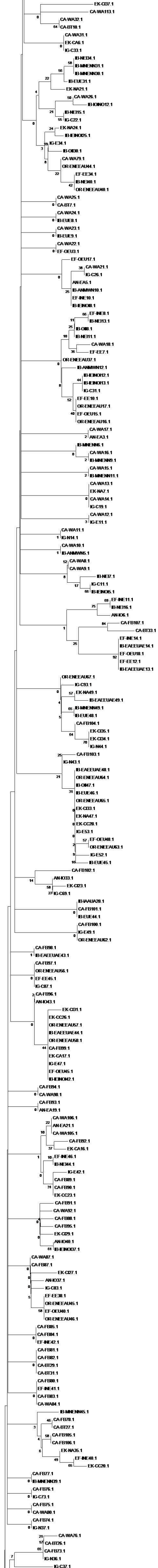

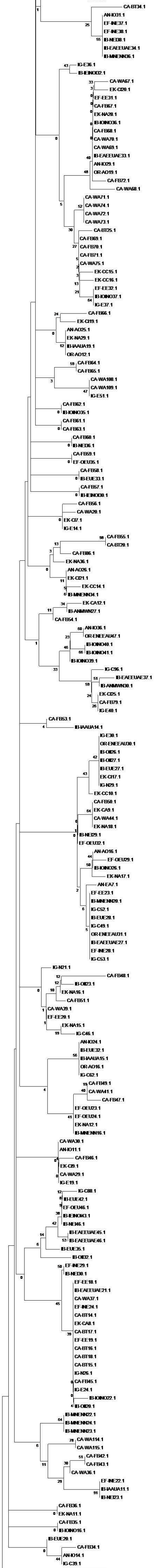

CA-BT34.1
AN-IO31.1
EF-INE37.1
EF-INE38.1
IB-NED8.1
IB-EAEEUAE34.1
IB-MNENN36.1
IG-E36.1
IB-IEINOD32.1
CA-WA67.1
EK-CI20.1
EF-EE31.1
CA-FB67.1
EK-NA28.1
IB-IOINO36.1
CA-FB68.1
CA-WA70.1
CA-WA69.1
IB-EAEEUAE33.1
AN-IO29.1
OR-AO19.1
CA-FB72.1
CA-WA68.1
CA-WA71.1
CA-WA74.1
CA-WA72.1
CA-WA73.1
CA-BT25.1
CA-FB69.1
CA-FB70.1
CA-FB71.1
CA-WA75.1
EK-CC15.1
EK-CC16.1
EF-EE32.1
IB-IOINO37.1
IG-E37.1
CA-FB66.1
EK-CI19.1
AN-AO25.1
EK-NA29.1
IB-IAAUA19.1
OR-AO12.1
CA-FB64.1
CA-FB65.1
CA-WA108.1
CA-WA109.1
IG-E51.1
CA-FB62.1
IB-IOINO35.1
CA-FB61.1
CA-FB63.1
CA-FB60.1
IB-NED6.1
CA-FB59.1
EF-OEU35.1
CA-FB58.1
IB-EUE33.1
CA-FB57.1
IB-IEINOD30.1
CA-FB56.1
CA-WA20.1
EK-CI7.1
IG-E14.1
CA-FB55.1
CA-BT20.1
CA-FB86.1
EK-NA36.1
AN-AO26.1
EK-CI21.1
EK-CC14.1
IB-MNENN34.1
EK-CA12.1
IB-ANMWN27.1
CA-FB54.1
AN-IO36.1
OR-ENEEAU47.1
IB-IOINO40.1
IB-IOINO41.1
IB-IOINO39.1
IG-C96.1
IB-EAEEUAE37.1
IB-ANMWN30.1
EK-CI25.1
CA-FB79.1
IG-E40.1
CA-FB53.1
IB-IAAUA14.1
IG-E30.1
OR-ENEEAU30.1
IB-OI26.1
IB-OI27.1
IB-EUE27.1
EK-CI17.1
IG-N29.1
EK-CC10.1
CA-FB50.1
EK-CA9.1
CA-WA44.1
EK-NA18.1
IB-NED29.1
EF-OEU32.1
AN-AO16.1
EF-OEU29.1
IB-IOINO26.1
EK-NA17.1
AN-EA7.1
EF-EE23.1
IB-MNENN20.1
IG-C52.1
IB-EUE28.1
IG-C49.1
OR-ENEEAU31.1
IB-EAEEUAE27.1
EF-INE28.1
IG-C53.1
IG-N21.1
CA-FB48.1
IB-OI23.1
EK-NA16.1
CA-FB51.1
CA-WA39.1
EF-EE20.1
EK-NA15.1
IG-C46.1
AN-IO24.1
IB-EUE32.1
IB-IAAUA15.1
OR-AO16.1
IG-C62.1
CA-FB49.1
CA-WA41.1
CA-FB47.1
EF-OEU23.1
EF-OEU24.1
EK-NA12.1
IB-MNENN16.1
CA-WA30.1
AN-IO11.1
CA-FB46.1
EK-CI9.1
CA-WA29.1
IG-E19.1
IG-C88.1
IB-EUE42.1
EF-OEU46.1
IB-IEINOI43.1
IB-NE46.1
IB-EAEEUAE45.1
IB-EAEEUAE46.1
IB-EUE35.1
IB-OI32.1
EF-INE29.1
IB-NED30.1
EF-EE18.1
IB-EAEEUAE21.1
CA-WA37.1
EF-INE24.1
CA-BT14.1
EK-CA8.1
CA-BT17.1
EF-EE19.1
CA-BT16.1
CA-BT18.1
CA-BT15.1
IG-N26.1
CA-FB45.1
IG-E24.1
IB-IOINO22.1
IB-OI20.1
IB-MNENN22.1
IB-MNENN24.1
IB-MNENN23.1
CA-WA114.1
CA-WA115.1
CA-FB42.1
CA-FB43.1
CA-WA36.1
EF-INE22.1
IB-IAAUA11.1
IB-NED23.1
CA-FB36.1
EK-NA11.1
CA-FB35.1
IB-IOINO16.1
IB-EUE20.1
CA-FB34.1
AN-IO14.1
IG-C39.1

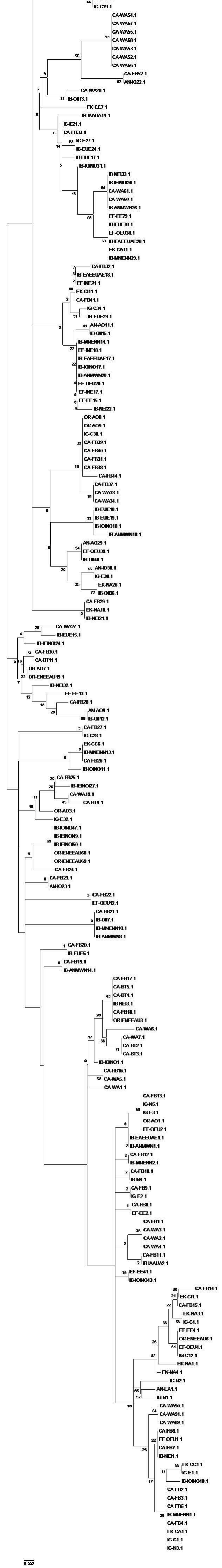

Cluster 4

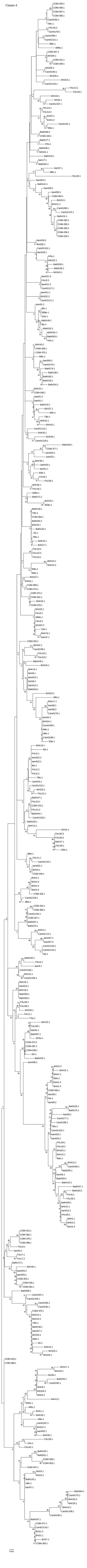

Cluster 5

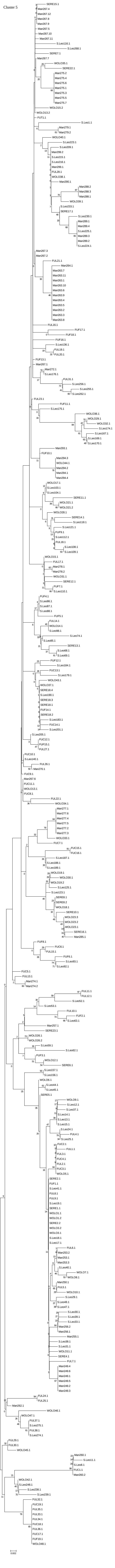

CLuster 6

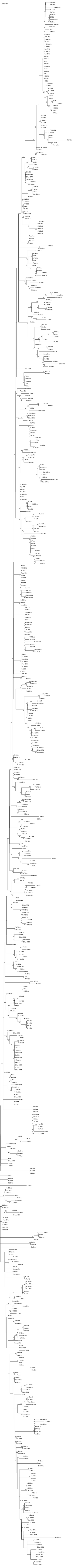

Cluster 7

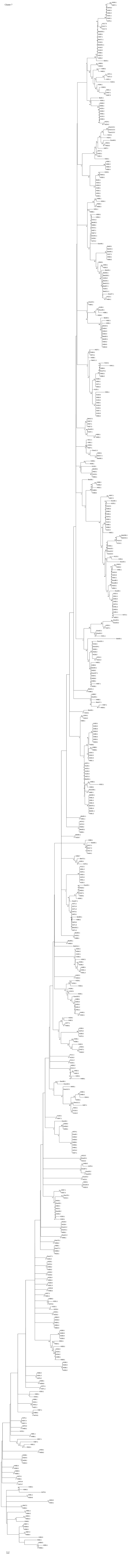

Supplement: Additional file 6: Figure S3 — Minimum Evolution tree topologies for the 7 DAPC clusters (see Supplementary Material for further details). The tree for cluster 3 was divided across 9 pages for a better graphical visualization. [file 1471-2148-13-24-S6.pdf]
